# Global spatial analysis of Arabidopsis natural variants implicates 5′UTR splicing of *LATE ELONGATED HYPOCOTYL* in responses to temperature

**DOI:** 10.1111/pce.13188

**Published:** 2018-04-15

**Authors:** Allan B. James, Stuart Sullivan, Hugh G. Nimmo

**Affiliations:** ^1^ Institute of Molecular, Cell and Systems Biology, College of Medical, Veterinary and Life Sciences University of Glasgow Glasgow G12 8QQ UK

**Keywords:** 5′UTR, alternative splicing, Arabidopsis, circadian clock, natural variation, RNA, temperature, thermosensor

## Abstract

How plants perceive and respond to temperature remains an important question in the plant sciences. Temperature perception and signal transduction may occur through temperature‐sensitive intramolecular folding of primary mRNA transcripts. Recent studies suggested a role for retention of the first intron in the 5′UTR of the clock component LATE ELONGATED HYPOCOTYL (LHY) in response to changes in temperature. Here, we identified a set of haplotypes in the LHY 5′UTR, examined their global spatial distribution, and obtained evidence that haplotype can affect temperature‐dependent splicing of LHY transcripts. Correlations of haplotype spatial distributions with global bioclimatic variables and altitude point to associations with annual mean temperature and temperature fluctuation. Relatively rare relict type accessions correlate with lower mean temperature and greater temperature fluctuation and the spatial distribution of other haplotypes may be informative of evolutionary processes driving colonization of ecosystems. We propose that haplotypes may possess distinct 5′UTR pre‐mRNA folding thermodynamics and/or specific biological stabilities based around the binding of trans‐acting RNA splicing factors, a consequence of which is scalable splicing sensitivity of a central clock component that is likely tuned to specific temperature environments.

## Abstract 2

How plants perceive and respond to temperature remains an important question in the plant sciences. We investigated the global spatial distribution of haplotypes for the 5′UTR region of the core circadian clock component LATE ELONGATED HYPOCOTYL (LHY) and obtained evidence that haplotype influences temperature‐dependent splicing of LHY transcripts. Our work suggests that scalable splicing sensitivity tunes the circadian clock to specific temperature environments.

## 1 INTRODUCTION

Temperature is a potent stimulus influencing plant morphology and reproductive development (McClung, Lou, Hermand, & Kim, 2016) and is on a par with the influential effects of light quantity and quality (Quint et al., 2016; Wigge, 2013). In Arabidopsis thaliana, temperature governs plant morphology and life history over a wide scale. Exposure to ambient temperatures that are high, but not heat stress inducing, influences elongation of the hypocotyl and flowering, referred to as thermomorphogenesis (Quint et al., 2016), whereas extended periods of cold, or vernalization, affect epigenetic modifications of key components of the flowering time pathway (Berry & Dean, 2015; Hepworth & Dean, 2015; Song, Irwin, & Dean, 2013).

Recent interest has focused on understanding how plants perceive and transduce temperature information to deliver physiological responses to temperature (Capovilla, Pajoro, Immink, & Schmid, 2015; McClung et al., 2016; McClung & Davis, 2010; Wigge, 2013). Although the identity of molecular plant thermometers has been described as “one of the great mysteries in the plant sciences” (McClung & Davis, 2010), recent studies using seedlings point to a role for phytochrome red light receptors as thermosensors (Jung et al., 2016; Legris et al., 2016); a role for the histone variant H2A.Z as a flowering time thermosensor (Kumar & Wigge, 2010); and roles for the phytochrome signalling component PHYTOCHROME INTERACTING FACTOR4 (Koini et al., 2009; Kumar et al., 2012), the circadian clock evening complex component EARLY FLOWERING3 (Box et al., 2015; Ezer et al., 2017), and CONSTANS (Fernandez, Takahashi, Le Gourrierec, & Coupland, 2016) in thermoresponsiveness. Additionally, temperature information may be routed via alternative splicing (AS) of components of several networks, including for the circadian clock (Calixto, Simpson, Waugh, & Brown, 2016; Filichkin et al., 2010; Filichkin et al., 2015; Filichkin & Mockler, 2012; James, Syed, Bordage, et al., 2012; James, Syed, Brown, & Nimmo, 2012; Kwon, Park, Kim, Baldwin, & Park, 2014; Seo et al., 2012), for light signalling components (Mancini et al., 2016; Shikata et al., 2014; Wu et al., 2014; Zhang, Lin, & Gu, 2017), and for flowering time (Capovilla, Symeonidi, Wu, & Schmid, 2017; Melzer, 2017; Pose et al., 2013; Sureshkumar, Dent, Seleznev, Tasset, & Balasubramanian, 2016). A prominent theme emerging from these advances is the role played by the circadian clock in integrating environmental cues (Arana, Tognacca, Estravis‐Barcala, Sanchez, & Botto, 2017; Ezer et al., 2017; Greenham & McClung, 2015), enabling endogenous clock rhythms to coincide with externally imposed cycles of light:dark and temperature (McClung et al., 2016; Nomoto et al., 2013; Yamashino, 2013).

The circadian clock enhances biological fitness by allowing organisms to anticipate environmental changes (Dodd et al., 2005; Green, Tingay, Wang, & Tobin, 2002). Its pace is largely unaffected across a range of physiologically relevant temperatures (Pittendrigh, 1960); this is termed temperature compensation and is usually studied by measuring circadian period at different fixed temperatures (Gould et al., 2006; Gould et al., 2013). In contrast to focusing solely on acclimated temperatures, we examined clock genes in mature Arabidopsis thaliana plants both during and after cooling. This identified temperature‐dependent AS in several genes including *LATE ELONGATED HYPOCOTYL* (*LHY*), *CIRCADIAN CLOCK ASSOCIATED1*, and *PSEUDO RESPONSE REGULATOR7* (James, Syed, Bordage, et al., 2012). The retention of the 5′UTR intron 1 in *LHY* (I1R, event UAS4 in James, Syed, Bordage, et al., 2012) and the inclusion of exon 5a (event AS5 in James, Syed, Bordage, et al., 2012) reach physiologically important levels in cooling and control LHY protein levels. Notably the former AS event is transient whereas the latter is adaptive to temperature (James, Syed, Bordage, et al., 2012; James et al., 2018). Conceptually, the regulation of *LHY* therefore represents an interesting model of how the clock adapts to (a) fluctuations and (b) longer term changes in temperature that are analogous in nature to unpredictable everyday changes and longer term (conceivably seasonal) changes in temperature, respectively. The *LHY* I1R event is of particular interest, because switching between fully spliced (FS) and I1R isoforms with temperature is rapid and reversible (James, Syed, Bordage, et al., 2012), has characteristics of a thermometer in that it is sensitive to temperature changes as modest as 2 °C, and is scalable and reversible over a wide dynamic range of temperature (James et al., 2018).

There is now clear evidence that pre‐mRNA secondary structure can influence the outcome of the splicing process (Buratti & Baralle, 2004; Ding et al., 2014; Gueroussov et al., 2017; Li et al., 2012; Soemedi et al., 2017; Vandivier, Anderson, Foley, & Gregory, 2016). Pre‐mRNA processing is simultaneous, and mechanistically coupled, to transcription with splicing factors (SFs) recruited either constitutively or on an “as needed” basis to intron‐containing genes (Bentley, 2014). The complexity of temperature signalling has recently been augmented with studies revealing cooling associated “splicing of the splicing factors” (Verhage et al., 2017; James et al., 2018), for example, for temperature‐associated isoform switching of the polypyrimidine (pY) tract‐binding (PTB) proteins and U2 auxiliary factor 65A (James et al., 2018) both of which compete for interaction with pY‐rich sequences thereby influencing efficiency of splicing (Simpson et al., 2014).

Natural variation of genes sensitive to temperature‐associated AS has yet to be characterized. However, there are several resources available to test correlations between sequence variations of candidate genes with climatic variables such as temperature. Whole genome sequencing of at least 1001 naturally inbred Arabidopsis lines, or accessions, led by The 1001 Genomes Consortium (http://1001genomes.org), has resulted in 1135 resequenced natural accessions that cover both the native Eurasian and North African range and recently colonized North America (Consortium, 2016). Using high‐resolution spatially interpolated climate data for global land areas, we mapped the distribution of four *LHY* 5′UTR haplotypes with a series of global bioclimatic variables and altitude, reasoning that if splicing of the *LHY* 5′UTR constituted a bona fide temperature sensing module, haplotype distribution would stratify with temperature climatic parameters.

We found that distinct haplotypes delineate along the lines of annual mean temperature and extent of annual temperature fluctuation (annual mean diurnal range) and that haplotype accessions are distinct in their temperature‐dependent splicing of *LHY* pre‐mRNA. We discuss these findings in the context of the transduction of temperature information to the clock via modulation of pre‐mRNA intramolecular folding and temperature scalable splicing sensitivity that is likely tunable to specific climatic regions.

## 2 MATERIALS AND METHODS

### 2.1 Sequence analysis and spatial analysis

The Arabidopsis 1001 Genome Browser (http://signal.salk.edu/atg1001/3.0/gebrowser.php) was used for preliminary analysis of single nucleotide polymorphism (SNPs) at the *LHY* locus. Sequence base calls for each of five SNP coordinates (Chr1: 37437, 37268, 37245, 37138, and 37072) for 1,135 accessions were obtained using the Pseudogenomes tool (http://tools.1001genomes.org/pseudogenomes/#select_strains). Latitude and longitude coordinates for the accession collection sites were also obtained (http://1001genomes.org/accessions.html). ADMIXTURE assignations for individual accessions were obtained from the 1001 Genomes Admixture map tool (http://1001genomes.github.io/admixture-map). Gviz (Hahne & Ivanek, 2016) was used to annotate *LHY* gene organization features with SNP frequencies. The sample() function in R was used to obtain random accession groups from the WRLD dataset. Maps were prepared using the map_data() function and a coord_fixed(1.3) aesthetic in ggplot2 in R. Bioclimatic variables at a global resolution of 2.5 arcmin, reflecting interpolations of observed data from 1960 to 1990, were obtained at http://worldclim.org (Hijmans, Cameron, Parra, Jones, & Jarvis, 2005). The global index maps were created by plotting the bioclimatic raster layer objects, with projected accession coordinates made explicitly spatial using the coordinates() function from the sp package in R and a coordinate reference system using the PROJ.4 spatial projection and the Earth shape reference datum WGS84. Bioclimatic variables were extracted for spatial locations using the extract() function from the raster package in R and represented mean values of raster cells in a 5‐km radius around each point location. Elevations, based on the latitude–longitude coordinates of the accession collection site, were obtained at http://www.gpsvisualizer.com/elevation.

### 2.2 RNA secondary structure prediction

*LHY* 5′UTR sequence of length 782 nt (−779 to +3 at the canonical start codon) was used in all RNA secondary structure prediction algorithms. R4RNA (Lai, Proctor, Zhu, & Meyer, 2012), using dot–bracket output of the RNA folding prediction web‐server mfold (Zuker, 2003) was used for RNA structure comparison analysis. Predicted thermodynamic stabilities (Gibbs free energy, Δ*G*, kcal/mol) of 5′UTR pre‐mRNAs were based on the folding algorithms of mfold (Zuker, 2003) and RNAfold, a component of the ViennaRNA Package 2.0 (Lorenz et al., 2011; http://rna.tbi.univie.ac.at/cgi-bin/RNAWebSuite/RNAfold.cgi). Secondary structure drawings were graphical output of RNAfold (Lorenz et al., 2011).

### 2.3 In vitro mRNA synthesis and labelling

High‐fidelity DNA Taq polymerase (Phusion, ThermoFisher Scientific) was used to amplify *LHY* from genomic DNA (isolated from Col‐0, DNeasy Plant kit, Qiagen) using the primers gLHY‐f1; 5′‐CACGTGTCGATCTGCGATGACTTC‐3′and gLHY‐r1; 5′‐TGTAGAAGCTTCTCCTTCCAATCGAAGC‐3′ that was then template for the amplification of *LHY* section −774 to +438 bp (relative to the translational start site; corresponding to coordinates Chr1: 37,835 to 36,624) using the primers LHY‐ex1‐f2; 5′‐GCTGAGATTGCTTCTGGCTTCT‐3′ and LHY‐ex5‐r; 5′‐CTTTGTGAAGAACTTTTGTGC‐3′. This PCR product was inserted into pCR4‐TOPO (ThermoFisher Scientific), sequence verified, and linearized with *Spe*I (Promega). The 5′‐capped in vitro synthesized RNA was prepared using the mMESSAGE mMACHINE kit (Ambion) using the T7 RNA polymerase site according to the manufacturer's protocol. RNA was purified and recovered with ammonium acetate followed by phenol:chloroform (1:1) extraction and isopropanol precipitation. RNA (approximately 70–400 ng) was labelled using the RNA 3′end biotinylation kit (Pierce) according to the manufacturer's instructions. Labelled RNA was purified (MEGAclear, ThermoFisher Scientific); glycogen and salt precipitated and diluted in elution solution (MEGAclear kit) to approximately 50 fmol/μl.

### 2.4 Preparation and purification of recombinant PTB1 protein

*PTB1* complementary DNA (cDNA) in pETM‐20 (gift of Professor John Brown, The James Hutton Institute, Dundee, UK) was subcloned via BspHI‐NotI into the pHS expression vector (Christie et al., 2012) to produce 7xHis‐Strep II‐SUMO PTB1 in Escherichia coli expression strain Rosetta BL21 (DE3) pLysS (Merck). Cells were grown in LB broth to an OD_600_ of 0.6 and induced with 100 μM isopropyl‐β‐D‐thiogalactopyranoside at 18 °C for 16 hr. Cells were collected by centrifugation at 3,500 *g* for 20 min and snap frozen in liquid nitrogen. Cells were thawed and resuspended in EB buffer containing 50 mM Tris–HCl pH 8.0, 500 mM NaCl, 20 mM imidazole, 1 mM phenylmethylsulfonyl fluoride (PMSF), and a protease inhibitor mixture (cOmplete EDTA‐free; Roche); lysed by sonication; and centrifuged at 143,000 *g* at 4 °C for 20 min. The supernatant was incubated in batch with 2 ml TALON SuperFlow (GE Healthcare), washed in EB buffer before eluting with 50 mM Tris–HCl pH 8.0, 150 mM NaCl, 250 mM imidazole, and 1 mM PMSF. Eluted protein was incubated in batch with 1 ml Strep‐Tactin Superflow Plus (Qiagen) and washed with 50 mM Tris–HCl pH 8, 150 mM NaCl, and 1 mM PMSF. PTB1 was released from the resin by incubation with the SUMO (ULP‐1) protease at 4 °C for 16 hr (Christie et al., 2012).

### 2.5 RNA electrophoretic mobility shift assay

RNA EMSA was performed using the Pierce Biotechnology LightShift Chemiluminescent RNA EMSA kit (ThermoFisher Scientific) essentially according to the manufacturer's instructions, but with minor modifications. EMSA binding reactions (20‐μl final volume) consisted of 10 mM HEPES pH 7.3, 100 mM KCl, 1 mM MgCl_2_, 1 mM dithiothreiotol, 0.25 μg/μL tRNA, and varying amounts of purified, recombinant PTB1 protein. The 3′ biotin labelled RNA (approximately 10 fmol per binding reaction, predenatured at 95 °C for 3 min and held on ice) were carried out at room temperature for 30 min. Glycerol (5% [*v*/v]) and loading dye (LightShift kit) were added to binding reactions and loaded on 6% polyacrylamide gels (40:1 acrylamide:bisacrylamide ratio in 50 mM Tris/50 mM Glycine [pH 8.3]) and run in the cold at 10 V/cm for 3.5 hr, similar to that described in (Clerte & Hall, 2009). For competition binding reactions an excess of the same, unlabelled RNA was preincubated (10 min at room temperature) with recombinant PTB1 before addition of the biotin‐labelled RNA. Gels were electro‐blotted onto Amersham Hybond‐N+ nylon membrane (GE Healthcare Life Sciences) in 50 mM Tris/50 mM Glycine (pH 8.3) at 30 mA for 1 hr at room temperature and transferred RNA cross‐linked at 120 mJ/cm^2^ (Crosslinker CL‐508, Syngene, Cambridge, England). Biotin‐labelled RNA:PTB1 protein complexes were detected using Pierce Biotechnology's Chemiluminescent Nucleic Acid Detection Module (ThermoFisher Scientific), according to the manufacturer's instructions, and exposed to X‐ray film (Medical X‐ray Blue, Carestream Health, Hertfordshire).

### 2.6 Plant material and growth conditions

Plant material was the Columbia (Col‐0) ecotype or natural variant accessions Lan‐0 (CS76539), Shigu‐2 (CS76374), Borsk‐2 (CS76421), Don‐0 (CS76411), and Vie‐0 (CS76418), Nottingham Arabidopsis Stock Centre. Plants were grown hydroponically as described previously (James et al., 2008) in environmentally controlled growth cabinets (Microclima, Snijders Labs, Tilburg, The Netherlands) at 20 °C in 12 hr light:dark cycles. White light intensity (100 ± 20 μE m^−2^ s^−1^) was provided by Sylvania Grolux F36 W/GRO fluorescent tubes. Plants were harvested 5 weeks after sowing (one biological repeat was RNA extracted from approximately 50–100 mg of pooled tissue from 9–13 mature plants per temperature condition/time point). Plants were harvested at dawn at 20 °C with cooling to 4 °C initiated 12 hr later at dusk, with subsequent samples at the next dawn (Day 1, 4 °C) and at Day 4, 4 °C, and at Day 8, 4 °C. Harvested tissue was immediately frozen in liquid nitrogen and stored at −80 °C until further use.

### 2.7 RNA extraction, cDNA synthesis, and qPCR

RNA extraction, cDNA synthesis, and qPCR were performed essentially as described previously (James et al., 2008; James, Syed, Bordage, et al., 2012). Briefly, total RNA was extracted with the RNeasy Plant Mini kit (Qiagen) and DNase treated (DNA‐free; Ambion). cDNA was typically synthesized from 1 μg of total RNA using random hexamers and SuperScriptII reverse transcriptase (ThermoFisher Scientific). qPCR reactions (1:100 dilutions of cDNA) were performed with Brilliant III SYBR Green QPCR Master Mix (Agilent) on a StepOnePlus (Fisher Scientific U.K. Ltd., Loughborough, U.K.) real‐time PCR system. The average Ct values for *PP2A* (At1g13320, primers PP2A‐f2; 5′‐TAACGTGGCCAAAATGATGC‐3′ and PP2A‐r2; 5′‐GTTCTCCACAACCGCTTGGT‐3′) was used as internal control for expression levels. Primers LHY‐ex1‐f2; 5′‐GCTGAGATTGCTTCTGGCTTCT‐3′, and LHY‐ex2‐ex1‐r; 5′‐GCAGCCAAAACCCTTGAGAGTA‐3′ were used to amplify constitutively spliced *LHY* 5′UTR transcripts and primers LHY‐ex1‐int1‐f; 5′‐GGCTACTCTCAAGGGTATAACAGTT‐3′ and LHY‐ex3‐ex2‐r; 5′‐GATTCTAGAGAAACCAAACGAATCC‐3′ were used to amplify transcripts retaining intron 1. The delta–delta Ct algorithm (Livak & Schmittgen, 2001) was used to determine relative changes in gene expression from two technical replicate assays.

### 2.8 Statistical analyses

Ordinary one‐way analysis of variance (one‐way ANOVA) with Brown–Forsythe summaries, post hoc Tukey–Kramer, and unpaired *t* tests with equal *SD* analyses were carried out in GraphPad Prism (version 6). For Tukey–Kramer, pairs of means grouped by a horizontal line were not significantly different from each other (*p* > .05). For *t* tests, threshold significance summaries were ***p = .0001 to .001, **p = .001 to .01, and *p = .01 to .05. Principal component analyses (PCAs) were carried out in R using prcomp by applying a log transformation to the continuous bioclimatic variables and employing “set center” and “scale.” =TRUE to standardize the variables. PCAs were visualized using ggbiplot employing “elipse” =TRUE where contours are drawn at the default 68% probability for each haplotype group.

## 3 RESULTS

**Figure 1:**
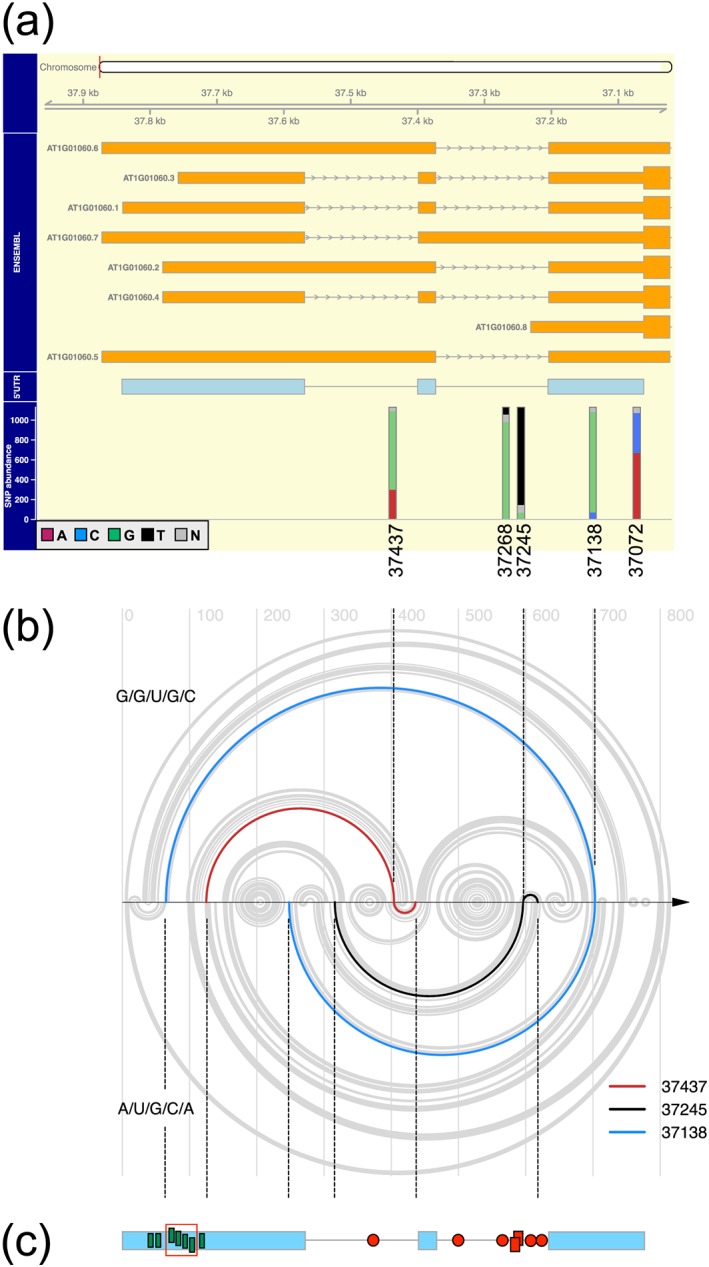
*LHY* 5′UTR haplotype prevalence and potential influence on pre‐mRNA secondary structure. (a) Single nucleotide polymorphism (SNP) coordinates and prevalence (%, vertical bars) for a set of 1,131 natural Arabidopsis variants. SNP bars align with ENSEMBL transcript models for the 5′UTR of *LHY* (At1g01060; orange assemblies) and the constitutively spliced model (At1g01060.1, pale blue model). Horizontal bars and lines; exons and introns, respectively. (b) R4RNA arc diagrams for predicted secondary structure comparison of the (upper) G/G/U/G/C and (lower) A/U/G/C/A haplotypes. Predictions are based on the folding of 782 nt (transcriptional start site to the first AUG start codon) of *LHY* pre‐mRNA. Vertical arrows and dotted lines map the coordinates of SNPs 37437, 37245, and 37138 from Panel (a) onto the arcs; coloured arcs highlight three SNP‐associated arcs. (c) Regions of secondary structure divergence for the three haplotypes in Panel (b) projected (vertical dotted lines) back onto the *LHY* 5′UTR model featuring red symbols, putative pY regions containing UCUU/UUCU (circles; regions <15 nt, rectangles regions >15 < 30 nt) and green rectangles, potential SUA consensus binding elements UCUUCUUC, including four tandem repeats (red outline). *LHY* = *LATE ELONGATED HYPOCOTYL*

We inspected the *LHY* locus (At1g01060) for SNPs and focused our attention on a subset of five SNPs (Figure S1, Figure 1a, and Table 1) located within the 5′UTR region because the balance between constitutive splicing and retention of the 5′UTR intron 1 in the *LHY* pre‐mRNA is scalable with temperature transitions (James, Syed, Bordage, et al., 2012; James et al., 2018). Sequence base calls, including uncalled N's, and latitude–longitude coordinates for each of the five SNP positions for 1,135 accessions were obtained (see Section 2). Four accessions (Table S1) were devoid of latitude–longitude information and were excluded from the dataset, as were accessions that possessed one or more uncalled “N” base. Inspection of this filtered dataset (our “WRLD” dataset, see Table S2 and the Supporting information Dataset file ‘LHY SNP WRLD dataset.csv’) revealed that 932 accessions each possessed one of four distinct 5′UTR haplotypes (Table 2). Col‐0, the Arabidopsis reference strain, possesses the G/G/U/G/C haplotype—the most prevalent *LHY* 5′UTR haplotype within the WRLD cohort.

**Table 1:** Single nucleotide polymorphism heterogeneity at the *LHY* 5′UTR locus (Chromosome 1; At1g01060) for 1,131 Arabidopsis thaliana accessions

Coordinate	Location	U	G	C	A	N
chr1:37437	intron 1	0	792	0	298	41
chr1:37268	intron 2	74	983	0	0	74
chr1:37245	intron 2	986	67	0	0	78
chr1:37138	exon 3	0	1013	70	0	48
chr1:37072	exon 3	0	0	404	668	59

*Note*. The number of natural variants from The 1001 Genome Consortium project (Consortium, 2016) possessing the indicated SNP at the denoted location in the genomic sequence for *LHY* is provided.

**Table 2:** Prevalence of haplotypes within the WRLD dataset

37437	37268	37245	37138	37072	Number (Proportion)
G	G	U	G	C	369 (39.6%)
G	G	U	G	A	320 (34.3%)
A	G	U	G	A	191 (20.5%)
A	U	G	C	A	52 (5.6%)

*Note*. Single nucleotide polymorphisms are ordered into four distinct haplotypes. The number of accessions and proportion of the 932 accessions (the WRLD dataset) used for global spatial analysis is provided.

**Figure 2:**
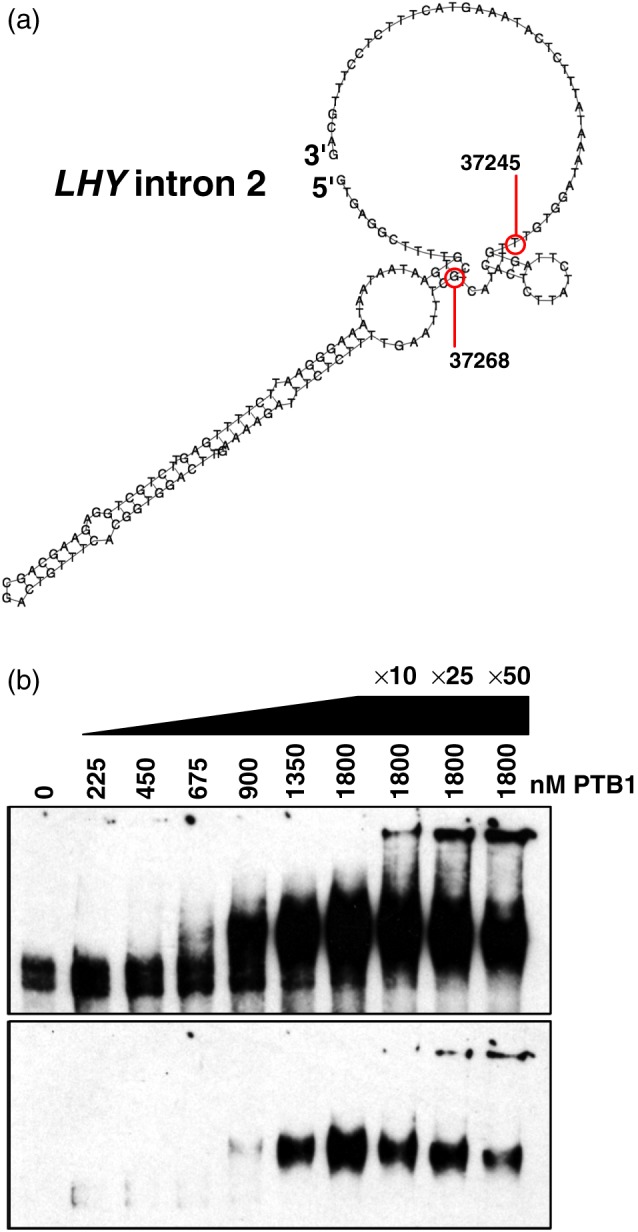
In vitro binding of PTB1 with *LHY* pre‐mRNA. (a) Predicted secondary structure (Vienna RNAfold) of intron 2 with location of SNPs 37245 and 37268 of the G/G/U/G/C haplotype highlighted. (b) In vitro RNA‐EMSAs (long and short exposures) showing the binding of recombinant PTB1 at the denoted concentrations with 3′‐biotin labelled *LHY* RNA (approximately 10 fg, spanning exons 1 to 5 plus the intervening introns), the three tracks on the right show competition with unlabelled RNA at the denoted fold abundance relative to labelled probe). *LHY* = *LATE ELONGATED HYPOCOTYL* [Colour figure can be viewed at http://wileyonlinelibrary.com]

We next compared pre‐mRNA secondary structure models for the four haplotypes. Table 3 summarizes the predicted contacts within the *LHY* 5′UTR made by the individual SNPs. Haplotype A/U/G/C/A displays a quite different pattern of base pairing compared to the other three haplotypes. We visualized the predicted structural differences between the Col‐0 haplotype, G/G/U/G/C, and A/U/G/C/A. To do this, we contrasted arc diagrams (R4RNA; Lai et al., 2012)—a method that uses the dot–bracket output annotation of RNA secondary structure algorithms such as mfold (Zuker, 2003). The arc diagram of Figure 1b points to the potential effects of variation at SNPs 37437, 37245, and 37138 to local and distant canonical base pairing. Notably, the 37245 and the 37138 SNPs of the G/G/U/G/C haplotype are predicted to make a local and a distant canonical base pair with regions enriched for PTB *cis*‐consensus sequence within a pY tract (Singh, Valcarcel, & Green, 1995; Wachter, Ruhl, & Stauffer, 2012) and SUA *cis*‐consensus sequences (Marquez, Hopfler, Ayatollahi, Barta, & Kalyna, 2015), respectively (Figures 1c and S2). These interactions are not evident for the A/U/G/C/A haplotype (Figure 1c) but are retained in the G/G/U/G/A and A/G/U/G/A haplotypes (Table 3). In addition, SNP 37268 is also predicted to make a different base pair in the A/U/G/C/A haplotype compared to the other three, though there is only a difference of 2 nts between contact sites. Interestingly, the thermodynamic stability (Gibbs free energy, Δ*G*, kcal/mol) of the G/G/U/G/C haplotype pre‐mRNA is more stable compared to the A/U/G/C/A haplotype (Table 4). Two SNPs—37268 and 37245—are located within intron 2 of the pre‐mRNA (Figure 1a), one of which (37268) is located at the neck of a region predicted to form a long (95 nt) stem loop (Figure 2a). Intron 2 is rich in PTB *cis*‐elements (Figure S2).

**Table 3:** Base pairing features of the SNPs at the 5′UTR region of *LHY*

Location	Coordinate 1	Pairing	Coordinate 2	Location
G/G/U/G/C				
exon 1	37716	C:G	37437	intron 1
intron 2	37360	U:G	37268	intron 2
intron 2	37223	A:U	37245	intron 2
exon 1	37776	C:G	37138	exon 3
	‐	‐:C	37072	exon 3
G/G/U/G/A				
exon 1	37716	C:G	37437	intron 1
intron 2	37360	U:G	37268	intron 2
intron 2	37223	A:U	37245	intron 2
exon 1	37776	C:G	37138	exon 3
	‐	‐:A	37072	exon 3
A/G/U/G/A				
	‐	‐:A	37437	intron 1
intron 2	37360	U:G	37268	intron 2
intron 2	37223	A:U	37245	intron 2
exon 1	37776	C:G	37138	exon 3
	‐	‐:A	37072	exon 3
A/U/G/C/A				
intron 1	37405	U:A	37437	intron 1
intron 2	37358	A:U	37268	intron 2
intron 1	37525	C:G	37245	intron 2
exon 1	37593	G:C	37138	exon 3
	‐	‐:A	37072	exon 3

*Note*. Single nucleotide polymorphism, as coordinate 2, location and mfold predicted association with Coordinate 1 canonical and non‐canonical pre‐mRNA base pair for each of the haplotypes. Dash (−) denotes non‐base pairing.

**Table 4:** Thermodynamic stability of *LHY* 5′UTRs with distinct haplotypes

Haplotype	Vienna RNAfold	mfold
A/G/U/G/A	−186.80	−175.60
G/G/U/G/A	−186.80	−175.46
G/G/U/G/C	−186.10	−175.46
A/U/G/C/A	−184.20	−173.56

*Note*. Vienna RNAfold and mfold predicted thermodynamic stabilities (Gibbs free energy, Δ*G*, kcal/mol) for 782 nts of *LHY* pre‐mRNA sequence (−779 to +3 relative to the ATG start codon) for each haplotype.

PTBs contribute to the temperature‐dependent splicing of *LHY* (James et al., 2018) and as such PTB would be predicted to interact specifically with *LHY* mRNA. To test this, we used RNA‐EMSA binding reactions with recombinant PTB1 protein and an in vitro transcribed *LHY* mRNA fragment comprising exons 1 through 5 and the intervening introns (representing the Col‐0 G/G/U/G/C haplotype) to ask whether PTB1 bound *LHY* pre‐mRNA. Figure 2b shows the formation of PTB1:*LHY* mRNA complexes that could be partially competed by the addition of excess unlabelled probe. The amount of probe bound seemed to increase cooperatively with the PTB1 concentration. Taken thus far, the structure predictions and binding data highlight that *LHY* 5′UTR splicing sensitivity is likely influenced by both the inherent thermodynamic stability (RNA secondary structure) of transcripts in addition to the biological stability of transcripts bound and processed by *trans‐*acting RNA‐binding SFs.

In order to investigate the potential relevance of the SNP variations to temperature‐dependent splicing of the 5′UTR of *LHY*, we next examined global spatial distribution of the Arabidopsis natural variants. The 932 accessions are distributed across 44 countries (Figure S3). Thirty countries, denoted as “others” in Figure S3a, have seven or less accessions (Figure S3b). Around 50% of the accessions in the WRLD dataset originate from Sweden, the Iberian Peninsula (Spain and Portugal), and the United States (Figure S3a). Differences in the relative make‐up of haplotypes within the country populations were evident—Recently colonized U.S. accessions (Hagmann et al., 2015) are principally the Col‐0‐like G/G/U/G/C type with a high proportion of the U.S. cohort (66%) originating from sites adjacent to Lake Michigan (Figure S4a). Other countries show increased stratification of haplotype distribution, as evident for the Spain and the U.K. populations (Figures S4b and S4e, respectively). Colonization of the British Isles is thought to have been more ancient and gradual compared to the United States (Consortium, 2016), and this is likely reflected in a wider spread of genotypes in the U.K. population compared to the United States. The latitudinal distribution of haplotypes in Spain (Figure S4b, inset), resolved with reasonably even sample sizes within 1° latitude “bins,” shows an increase in the proportion of the G/G/U/G/A haplotype with increasing latitude at the expense of the G/G/U/G/C and A/U/G/C/A haplotypes. Sweden provides the largest single contribution (23.5%) of accessions in the WRLD dataset (Figure S3a), primarily split between two sites—a northern “High Coast” group and a southern “Skåne” population (Figure S4c). These two regions display contrasting climates. The northern accessions, the majority of which are the G/G/U/G/A type (Figure S4c, inset), experience colder temperatures, longer snow cover, and a broader range of photoperiod, whereas the more stratified southern strains are found in agricultural meadows, fields, and on beaches along the Baltic Sea (Brachi, 2014).

Sample sizes for each haplotype within the WRLD dataset were uneven due to the relative rarity of A/U/G/C/A (Figure S5a). We therefore prepared a dataset (“WRLD_ran50”, see Supporting information Dataset file ‘LHY SNP WRLD_ran50.csv’) with equal haplotype sample sizes by randomly selecting 50 accessions for each of the four haplotypes from the original WRLD dataset (Figure S5a). The WRLD dataset contained a relatively high proportion (40%) of lines originating from the same latitude–longitude coordinates, but with the WRLD_ran50 dataset, this sample site redundancy was reduced to 11.5% (Figure S5b).

**Figure 3:**
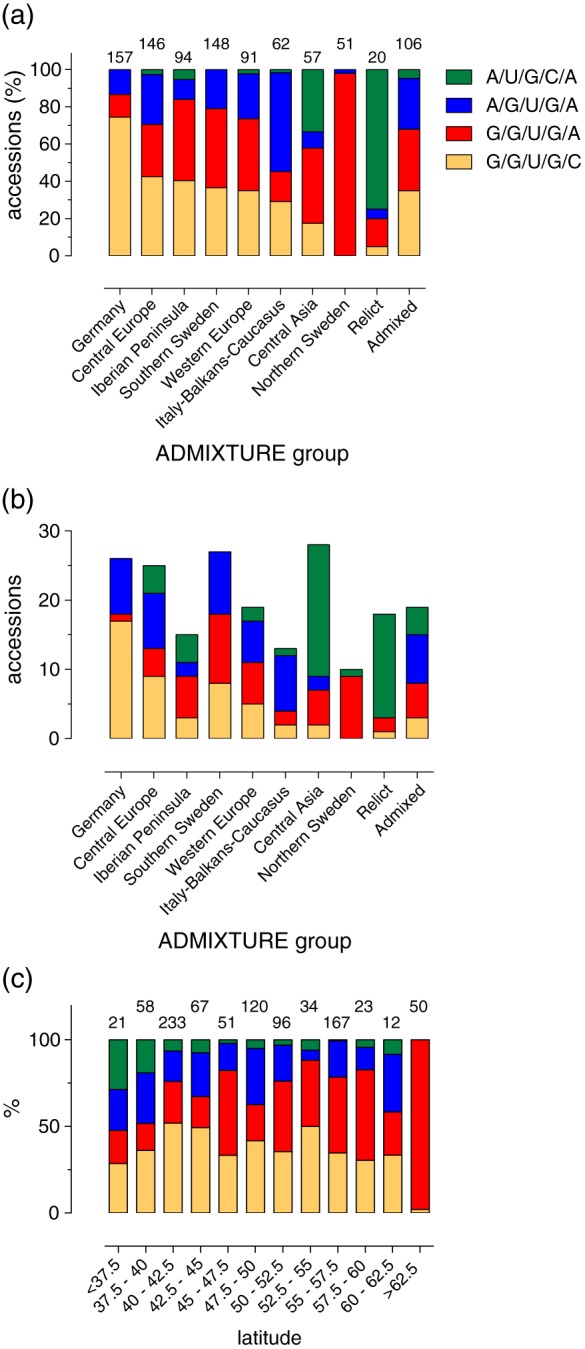
Global distribution of haplotypes. Frequency of haplotypes at the ADMIXTURE group level for the (a) WRLD and (b) WRLD_ran50 datasets. (c) Frequency of haplotypes at the latitude level. Colour shading categorization of haplotypes in Panels (b) and (c) is as Panel (a). For Panels (a) and (c), the number of accessions in each group is denoted above the bars [Colour figure can be viewed at http://wileyonlinelibrary.com]

Previously, we distributed the *LHY* 5′UTR haplotypes at the country level (Figures S3 and S4). However, The 1001 Genomes Consortium established that genetic distances between individual accessions did not reflect geographic distance (Consortium, 2016). We therefore next sought to associate haplotypes according to their ADMIXTURE assignations, a model‐based assessment of the ancestry of unrelated individuals (Alexander, Novembre, & Lange, 2009; Consortium, 2016). The clusters broadly correspond to geography (eight groups) and extreme ancestral divergence (the “relict” and “admixed” clusters). The distribution of the different haplotypes within these ADMIXTURE groups is shown in Figure 3a for the WRLD dataset and in Figure 3b for the WRLD_ran50 dataset. These data show representation of the haplotypes, albeit with different frequencies, within the majority of these clusters (Figure 3a)—the exception being the Northern Sweden cohort that is almost universally the “G/G/U/G/A” haplotype (Figure 3a). A similar distribution of haplotypes within the ADMIXTURE clusters for the WRLD_ran50 dataset was also seen (Figure 3b), suggesting that sampling accessions randomly across the world maintained an adequate representation of the full dataset. There was not a clear correlation of haplotype prevalence across the latitude range (Figure 3c), although interpretation may be hampered by sample size variation in each of the latitude “bins.”

Relict accessions are variants that continue to inhabit ancestral habitats and are thought to have mixed with other lineage during a spread to northern latitudes. The 1001 Genomes Consortium identified 22 Iberian relicts, 17 of which are present in our WRLD_ran50 datasets (20 are within the WRLD dataset; Table S2, Table S3, and Figure S4d). The majority of these are the A/U/G/C/A type (15 out of 17, and 15 out of 20 for WRLD_ran50 and WRLD, respectively). This haplotype therefore seems to be of relict origin—It is relatively rare (around 5% in the WRLD dataset [Table 2] and 9% in WRLD_ran50 dataset [Table S2]) and, as noted earlier, probably possesses distinct secondary structure properties compared to the more prevalent haplotypes (Figure 1b and Tables 3 and 4).

**Figure 4:**
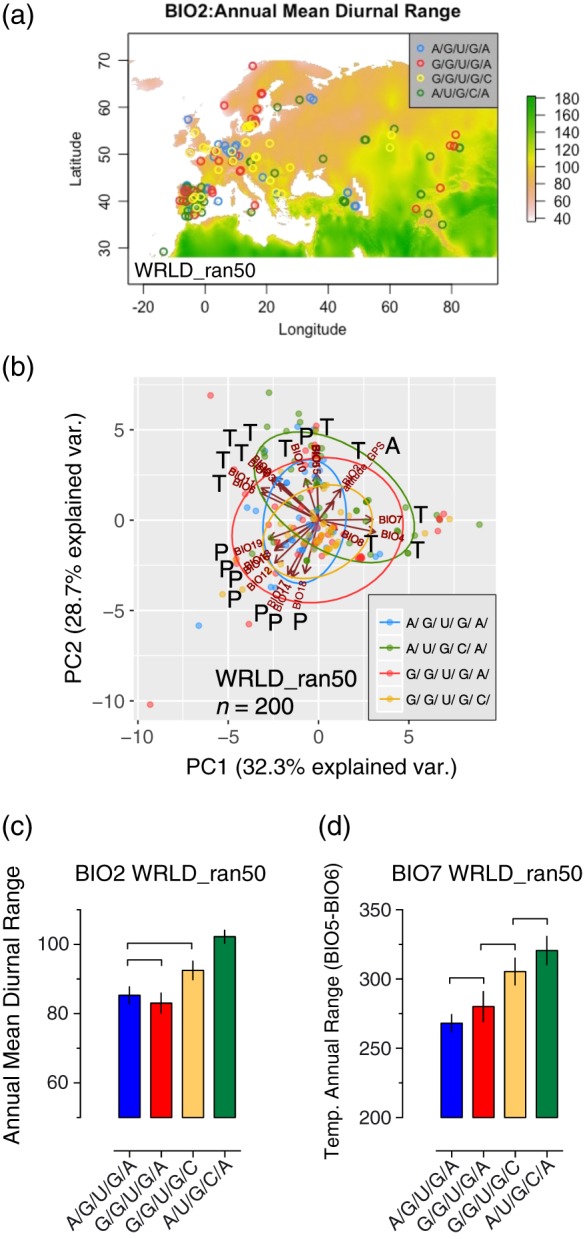
Haplotypes correlate with bioclimatic variables. (a) Projection of haplotypes from the WRLD_ran50 dataset onto the global index of annual mean diurnal range (BIO2, http://worldclim.org, index scale; °C × 10). Accessions are plotted across Europe, North Africa, and Central Asia. A global projection of the entire WRLD_ran50 dataset is presented in Figure S6a. (b) Principal component analysis (PC1 vs. PC2) of 20 continuous variables (11 temperature [T], 8 precipitation [P], and 1 altitude [A] variable) and categorized for the four haplotypes for the WRLD_ran50 dataset. Means and ±SEM of (c) BIO2: annual mean diurnal range (°C × 10) and (d) BIO7: temperature annual range (°C × 10) for each haplotype cohort. Pairs of means grouped by a horizontal bracket are not significantly different from each other (Tukey–Kramer method, *p* > .05; see Section 2) [Colour figure can be viewed at http://wileyonlinelibrary.com]

We next investigated correlations between *LHY* 5′UTR haplotype distribution with a series of 19 bioclimatic variables at a global resolution of 2.5 arcmin (http://worldclim.org; Hijmans et al., 2005). These variables included monthly total precipitation, and monthly mean, minimum, and maximum temperature, and another 15 variables derived from the monthly data. Elevations for the accession collection sites were also obtained (see Section 2). Figure 4a shows the WRLD_ran50 dataset accessions mapped upon global means of monthly temperature ranges (BIO2: annual mean temperature range), an index useful for interpreting the relevance of temperature fluctuation to a species (O'Donnell & Ignizio, 2012). Figure S6a shows an equivalent plot for the 932 accessions of the WRLD dataset mapped upon BIO2 variables. The WRLD and WRLD_ran50 datasets (Table S2) contain accession details, *LHY* 5′UTR haplotype and ADMIXTURE categorization and latitude–longitude, bioclimatic and elevation information.

PCA was next used to visualize variation between the 20 continuous (bioclimatic and elevation) variables and the four categorical (haplotype) variables for the WRLD_ran50 and WRLD datasets (Figures 4b and S6b, respectively). For both datasets, six principal components could explain around 96% of the variation of the continuous variables (Figure S7). The PCAs show clustering of the precipitation and temperature variables (denoted as “P” and “T” in the plots) with the principal component dimensions. Although these data show a high degree of overlap between the haplotypes, it seems that at least the G/G/U/G/A haplotype diverged from the A/U/G/C/A haplotype across the PC2 (Figures 4b and S6b). We focused on the nonredundant WRLD_ran50 dataset, reasoning that a balanced design (equal number of observations in each haplotype group) would minimize heteroscedasticity (different standard deviations in the different groups), a consideration for ANOVA interpretations. We selected bioclimatic variables meriting further analysis based on (a) an ANOVA assessment of the variability among haplotype group means and (b) a test of the assumption that the group variances were statistically equal (Brown–Forsythe test; Table 5). On this basis, clinical correlations between the categorical haplotype variables and temperature bioclimatic variables can be seen—especially for BIO2 (mean diurnal range) and BIO7 (temperature annual range; Table 5). Post hoc multiple comparison tests (Tukey–Kramer; confidence interval for comparisons of means with every other mean) were performed for BIO2 and BIO7 for both the WRLD_ran50 and WRLD datasets. These data show that the A/U/G/C/A haplotype correlates with a higher extent of annual temperature fluctuation (BIO2; Figures 4c and S6c for the WRLD_ran50 and WRLD datasets, respectively), and a higher temperature difference between the minimum temperature of the coldest month and the highest temperature of the warmest month (BIO7; Figures 4d and S6d). The previously noted 20 relict accessions in the WRLD dataset (Tables S2 and S3) are principally A/U/G/C/A (15 out of 20 accessions). Plots of the individual relict accession BIO2 and BIO7 levels indicate that the A/U/G/C/A relicts are not responsible for the higher mean levels of the A/U/G/C/A BIO2 and BIO7 variables compared to other haplotypes (Figure S6c,d, respectively). Haplotypes G/G/U/G/C and G/G/U/G/A differ—at least for the 5′UTR region analysed—only in the SNP adjacent to the translational start site (37072), yet G/G/U/G/C accessions associate with wider fluctuations in temperature (Figure 4c) and wider extremes of temperature (Figure 4d). A/G/U/G/As and G/G/U/G/As, which differ only in SNP 37437, share similar responses to fluctuations (i.e., the extent of habitat temperature “bandwidth”; Figure 4c) yet appear to be geared to environments with distinct annual mean temperatures (BIO1; Figure S8).

**Table 5:** ANOVA and Brown–Forsythe statistics for the WRLD_ran50 dataset

WORLDCLIM Description	Abbrev.	Type	ANOVA *F*_3,196_	Brown–Forsythe	B–F significance
Annual mean temperature	BIO1	T	=2.88, *p* = .037*	*p* < .0001****	Y
Mean diurnal range	BIO2	T	=11.98, *p* < .0001****	*p* = .016*	Y
Isothermality (BIO2/BIO7) (* 100)	BIO3	T	=2.20, *p* = .089	*p* = .015*	Y
Temp. seasonality (st. dev. *100)	BIO4	T	=3.72, *p* = .012*	*p* = .005**	Y
Max. temp. of warmest month	BIO5	T	=4.94, *p* = .003**	*p* = .722	N
Min temp. of coldest month	BIO6	T	=3.53, *p* = .016*	*p* < .0001****	Y
Temp. annual range (BIO5‐BIO6)	BIO7	T	=6.34, *p* = .0004***	*p* = .051	N
Mean temp. of wettest quarter	BIO8	T	=5.50, *p* = .001**	*p* = .120	N
Mean temp. of driest quarter	BIO9	T	=2.25, *p* = .084	*p* = .0005***	Y
Mean temp. of warmest quarter	BIO10	T	=3.16, *p* = .016*	*p* = .320	N
Mean temp. of coldest quarter	BIO11	T	=2.66, *p* = .050	*p* < .0001****	Y
Annual precipitation	BIO12	P	=4.79, *p* = .003**	*p* = .250	N
Precipitation of wettest month	BIO13	P	=0.86, *p* = .461	*p* = .838	N
Precipitation of driest month	BIO14	P	=10.72, *p* < .0001****	*p* = .915	N
Precipitation seasonality (CV)	BIO15	P	=8.24, *p* < .0001****	*p* = .461	N
Precipitation of wettest quarter	BIO16	P	=1.36, *p* = .225	*p* = .630	N
Precipitation of driest quarter	BIO17	P	=10.33, *p* < .0001****	*p* = .779	N
Precipitation of warmest quarter	BIO18	P	=12.43, *p* < .0001****	*p* = .744	N
Precipitation of coldest quarter	BIO19	P	=0.88, *p* = .451	*p* = .129	N
Elevation	altitude_GPS	‐	=17.22, *p* < .0001****	*p* = .0002***	Y

*Note*. A test of the null hypothesis that the haplotype group means were identical for each of the WORLDCLIM bioclimatic variables (Hijmans et al., 2005) and the elevation variable was tested by ordinary one‐way ANOVA. For the null hypothesis to be true the *F* ratio value is expected to be close to 1.0 and a large *F* ratio indicates that the variation among haplotype group means is more than expected by chance. A small *p* value (in the ANOVA column) indicates that it is unlikely that the differences observed are due to random sampling. Bioclimatic variables are summarized as either temperature (“T”) or precipitation (“P”) associated. The Brown–Forsythe test is reported as a *p* value and indicates whether the haplotype group populations have different standard deviations and is summarized at a significance level (“B–F significance”) as either yes (“Y”) or no (“N”) (*p* < .05). ANOVA = analysis of variance.

Taken together, a general picture emerges of haplotype temperature “specialisms”—Although A/U/G/C/As and G/G/U/G/As both tend to associate with cooler environments (BIO1; Figure S8), A/U/G/C/As are prominent in environments with wider extremes of temperature (BIO7; Figure 4d). On the other hand, A/G/U/G/As and G/G/U/G/Cs both appear to correlate with milder temperatures (BIO1; Figure S8), with G/G/U/G/Cs tending to associate with wider maximum–minimum temperature habitats (BIO2; Figure 4c).

A/U/G/C/As are notably distinct from the other three haplotypes for precipitation bioclimatic variables (Table 5 and, e.g., BIO14, BIO17, and BIO18 in Figure S9). A similar picture is seen for the annual precipitation variable (BIO12, Figure S10) where A/U/G/C/A is clearly distinct from the other haplotypes. Interestingly, scatter plots of BIO1 (annual mean temperature) with BIO12 (annual precipitation) imply that for Iberian and Swedish populations (Figures S11a,b, respectively), the G/G/U/G/A haplotype has expanded into low temperature and high precipitation environments, whereas G/G/U/G/C haplotypes appear to occupy habitats with narrower ranges of temperature and precipitation.

**Figure 5:**
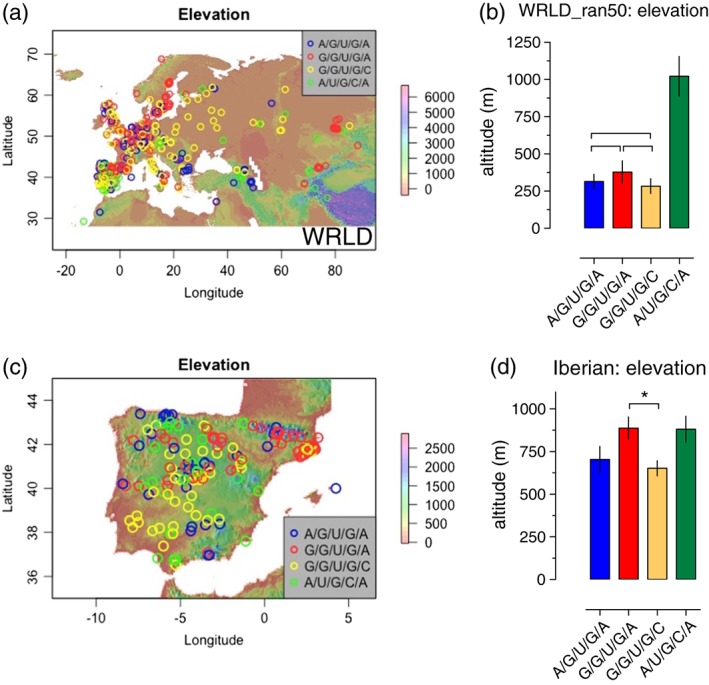
Haplotypes correlate with elevation. (a) Projection of haplotypes from the WRLD dataset onto the global elevation profile (index; metres). Accessions are plotted across Europe, North Africa, and Central Asia. (b) Means and ±SEM of elevations for each haplotype group for the left; WRLD_ran50 and right; WRLD datasets. Pairs of means grouped by a horizontal bracket are not significantly different from each other (Tukey–Kramer method, *p* > .05). (c) Left, projection of haplotypes onto the Iberian Peninsula elevation profile (index; metres), and right, means of elevations for each haplotype group for the Spanish cohort; all means not significantly different from each other (Tukey–Kramer method, *p* > .05), except for G/G/U/G/A versus G/G/U/G/C (Tukey–Kramer method, *p* < .05; see Section 2) [Colour figure can be viewed at http://wileyonlinelibrary.com]

The Quaternary glacial history of the Mediterranean Basin has played an important role in structuring patterns of plant biodiversity. Consequently, the Iberian Peninsula has emerged as an important backdrop for studying the evolutionary processes underlying plant differentiation (Comes & Kadereit, 1998; Hewitt, 1999; Hughes, Woodward, & Gibbard, 2006; Marcer et al., 2017; Médail & Diadema, 2009). There are also high levels of environmental heterogeneity in the region, leading to, for example, large differences in geographic variances of minimum temperature between the north and south of the Peninsula, and these appear to correlate strongly with Arabidopsis life cycle phenology (Marcer et al., 2017). Central and northwest Spain are mountainous areas with strong altitudinal gradients and rapid changes in ecological conditions over short distances. These mountainous areas have cool climates that are largely absent in the south‐west of the Iberian Peninsula (Mendez‐Vigo, Pico, Ramiro, Martinez‐Zapater, & Alonso‐Blanco, 2011). We were therefore interested to see the global distribution of the four haplotypes for the elevation variable in the WRLD dataset (Figures 5a and S6e) and also for the WRLD‐ran50 datasets (Figure 5b). These data show a global‐wide high altitude distribution of the relict A/U/G/C/A haplotype compared to the other three haplotypes. Iberian A/U/G/C/As also demonstrated a tendency for a higher altitude distribution (Figure 5c,d). Similar to that observed previously for the BIO2 and BIO7 variables, A/U/G/C/A accessions classified as relict by ADMIXTURE did not appear to influence the higher altitude distribution compared to the other haplotypes (Figure S6e).

The A/U/G/C/A haplotype was not the only high altitude specialist; there also seemed to be a prevalence for the G/G/U/G/A haplotype at higher elevations in the Iberian cohort (Figure 5d). Thus, there appears to be a tendency for low altitude, low latitude Iberian accessions to be G/G/U/G/C variants with higher altitude and higher latitude types to be of the G/G/U/G/A type (Figure S4b inset and Figure 5c,d). G/G/U/G/As seem to be particularly prevalent in the mountainous Pyrenees region of northern Spain, where there is a lower prevalence of relict A/U/G/C/As (Figure S4b inset and Figure 5c).

**Figure 6:**
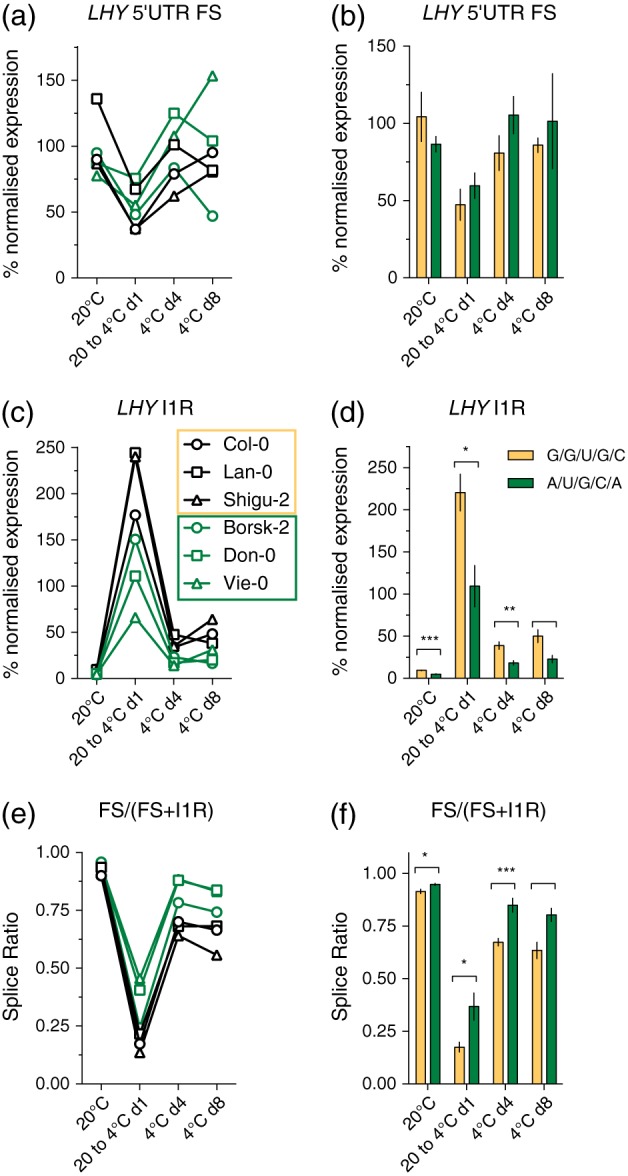
Distinct *LHY* 5′UTR splicing sensitivity for two haplotypes. Expression levels, at dawn for the denoted temperature conditions, for the individual accessions (Panels (a), (c), and (e)) and means and ±SEM for the grouped haplotypes (Panels (b), (d), and (f); *n* = 3) for ((a) and (b)) fully spliced (FS) 5′UTR, ((c) and (d)) intron 1 retained (I1R) transcripts, and ((e) and (f)) the splice ratio (FS transcripts as a fraction of total transcripts). Expression levels for individual accessions are derived from pooled tissue (9–13 plants per temperature condition) and from two technical repeats of the qPCR assay (see Section 2) [Colour figure can be viewed at http://wileyonlinelibrary.com]

We next asked whether the predicted transcript secondary structure of the *LHY* 5′UTR might correlate with its splicing response to cooling. We chose to compare representative accessions for two of the haplotype subgroups—the relict‐like A/U/G/C/A and the Col‐0‐like G/G/U/G/C subgroups—for which the bases in pre‐mRNA at positions 37268 and 37245 make different contacts (Figure 1b). Three accessions each for the two haplotypes (Figure S12) were subjected to cooling from 20 to 4 °C, with cooling initiated at dusk (see Section 2), and representative dawn phased samples for transition (Day 1) and acclimation (Days 4 and 8) to the lower temperature analysed. Levels of FS 5′UTR transcripts and transcripts retaining intron 1 (I1R transcripts) were determined (see Section 2). Previous work had demonstrated that FS:I1R levels, at dawn at 20 °C for Col‐0, was approximately 0.9 (James, Syed, Bordage, et al., 2012), and this ratio was therefore used as the reference against which all other accessions were compared. FS levels for the two subgroups were largely similar, with a tendency for G/G/U/G/Cs to have lower levels post‐cooling compared to A/U/G/C/As (Figure 6a,b). More strikingly, however, relict accessions appear to splice a lower proportion of transcripts to I1R under all conditions (Figure 6c,d), with the concomitant result that the splice ratio—the proportion of FS as a fraction of total levels—for relicts was higher compared to G/G/U/G/Cs (Figure 6e,f). This suggests that haplotype does indeed affect the splicing of *LHY* transcripts in response to cooling.

## 4 DISCUSSION

The circadian clock integrates multiple environmental stimuli, or “inputs,” with physiologically relevant “output” processes, and it is now well appreciated that there are fitness costs in running a dysfunctional clock (Dodd et al., 2005; Greenham & McClung, 2015; Yerushalmi & Green, 2009). The clock is keenly tuned to alternating light:dark and temperature cycles and is said to “gate” responsiveness to the environment to particular phases of the day via precise timing—or phasing—of individual clock components and their cognate downstream signalling cascades (Fowler, Cook, & Thomashow, 2005; Hotta et al., 2007). Large portions of the Arabidopsis transcriptome are clock controlled including growth, stress responses, hormone signalling, and metabolism pathways (Covington, Maloof, Straume, Kay, & Harmer, 2008; Harmer et al., 2000). One example is circadian clock gating of the cold response via the C‐REPEAT BINDING FACTOR regulon—comprising around 100 or so cold‐responsive genes—many of which contain *CIRCADIAN CLOCK ASSOCIATED1*/LHY *cis*‐binding elements within their promoters (Mikkelsen & Thomashow, 2009; Thomashow, 1999). Cooling also signals to the clock—trough levels of daily clock gene oscillations appear to rise with cooling resulting in damped rhythms (Bieniawska et al., 2008)—and cooling promotes temperature‐associated AS of several clock genes (Filichkin & Mockler, 2012; James, Syed, Bordage, et al., 2012; James, Syed, Brown, & Nimmo, 2012; Kwon et al., 2014; Seo et al., 2012). Precisely how temperature information is perceived and transduced to the clock via post‐transcriptional mechanisms is an area of speculation and how this might mechanistically coordinate gating of output pathways is not known. Similarly, it remains unclear to what extent temperature‐associated clock AS is mechanistically linked to inherently important core clock phenomena such as temperature compensation or temperature entrainment of the clock (Edwards et al., 2006; Edwards, Lynn, Gyula, Nagy, & Millar, 2005; Salome & McClung, 2005), although it is notable that splicing related components such as *SICKLE*, *GEMIN2*, and *SKIP* are implicated in temperature compensation (Marshall, Tartaglio, Duarte, & Harmon, 2016; Schlaen et al., 2015; Wang et al., 2012).

We based this analysis of 1,001 genomes accessions on our recent observations of the dynamic responses of *LHY* AS to temperature, and in particular, the “molecular thermostat” properties of 5′UTR splicing—an apparently adaptive response to temperature such that I1R splicing is most prominent during temperature transitions (and not “steady state” temperatures), akin to fluctuations in temperatures that are a hallmark of natural climatic conditions (James et al., 2018). We focused our attention on five SNPs in the 5′UTR region—two exonic and three intronic—that cluster as four haplotypes. We characterized the A/U/G/C/A haplotype, common to “relict” accessions, as the most distinct of the haplotypes in the respect that, worldwide, these accessions are found in regions of low rainfall. They are also associated with the highest elevations with low mean annual temperatures and a wider range of maximum–minimum temperatures. Two of the remaining three haplotypes seem to associate with milder annual mean temperatures (A/G/U/G/As and G/G/U/G/Cs) and lower altitude and wetter habitats. Interestingly, G/G/U/G/As seem to be a low temperature “specialist”—This haplotype is commonly found in the mountainous Pyrenees region of northern Spain and is prominent at the limit of Arabidopsis growth in northern Sweden.

It is not known whether the splicing of *LHY* transcripts is affected by water status, but it is clearly affected by temperature changes. Our data show that the splicing of the *LHY* 5′UTR on cooling does differ between a small number of representative “relict‐like” A/U/G/C/A and ‘Col‐0 like’ G/G/U/G/C accessions, consistent with the notion that the *LHY* 5′UTR represents a bona fide thermometer that is likely finely tuned to distinct temperature habitats. However, further work will be required to determine if other differences between the haplotypes contribute to these results. The 5′UTR is critical for ribosome recruitment to mRNAs and start codon choice plays a major role in the control of translation efficiency (Hinnebusch, Ivanov, & Sonenberg, 2016). It is currently unclear whether I1R transcripts retain translation potential and if so whether upstream open reading frames (uORFs) would play a role in fine‐tuning translational control of *LHY*. Equally, I1R transcripts might be devoid of translation potential and subject to degradation via nonsense mediated decay (NMD; Kalyna et al., 2012; Staiger & Brown, 2013; Syed, Kalyna, Marquez, Barta, & Brown, 2012). In this scenario, the pool of translatable message is likely compromised with a possibility that this results in reduced levels of LHY protein. Either way, LHY protein levels appear to be precisely set at dawn via prior post‐transcriptional regulation.

The importance of intron‐mediated regulatory mechanisms and the control of gene expression levels are increasingly recognized. The classic example of intron‐mediated regulation in plants is the epigenetic‐mediated regulation of *FLOWERING LOCUS C* (*FLC*) levels in “over‐wintering” plants (Song et al., 2013). Here, vernalization‐induced changes at the *FLC* locus in Arabidopsis occur specifically within intron 1, resulting in progressive gene silencing to enable competency to flower when suitable temperatures prevail. In cereals, the vernalization response is mediated by the stable induction of *VERNALIZATION1* (Greenup, Peacock, Dennis, & Trevaskis, 2009; Oliver, Finnegan, Dennis, Peacock, & Trevaskis, 2009) and natural variation in intron 1 length in *VERNALIZATION1* seems to modulate vernalization sensitivity in a manner that reflects Spring and Winter flowering time habits (Szucs et al., 2007). Regulation of the MADs box *FLOWERING LOCUS M* (*FLM*) component of the ambient temperature flowering pathway also appears to be regulated by an intron‐associated mechanism. In vernalization‐insensitive Arabidopsis accessions flowering in ambient temperatures is largely under control of the precise balance of FLM α‐ and β‐alternatively spliced isoforms (Capovilla et al., 2017; Pose et al., 2013; Sureshkumar et al., 2016) and natural variation within intron 1 of *FLM* results in varying splicing sensitivities that likely are advantageous for flowering time adaptation in the ambient temperature range (Lutz et al., 2015). Here, the primary sensing mechanism feeding into *FLM* splicing is unknown, but as with *LHY* splicing may conceivably involve the perturbation of a network of temperature‐associated isoform switching RNA binding proteins that include PTB1, U2 auxiliary factor 65A, and SUA (James et al., 2018).

At this stage, we cannot rule out the possibility that other SNPs cosegregate with the *LHY* SNPs to provide the correlation patterns presented here, and future work will require the assessment of the influence of *LHY* SNPs for temperature‐associated splicing sensitivity in isogenic or near isogenic backgrounds. We conclude that *LHY* 5′UTR haplotypes—possessing distinct pre‐mRNA folding stabilities and/or biological stabilities display a range of temperature specialisms that may have enabled Arabidopsis to colonize new temperature habitats. Given that global climate change is likely to have major but unpredictable effects on plant diversity and crop yields (Chakraborty & Newton, 2011; Hatfield et al., 2014; McClung & Davis, 2010; Moore & Lobell, 2015; Mora et al., 2015; Thuiller, Lavorel, Araujo, Sykes, & Prentice, 2005; Wheeler & von Braun, 2013), insights as to how plants perceive and integrate temperature information via the clock to physiologically relevant outputs and how evolution drives innovations in plants responses to temperature is likely of value to enhanced crop breeding programs.

## CONFLICT OF INTEREST

The authors declare no conflict of interest.

## AUTHOR CONTRIBUTIONS

A.B.J. and H.G.N. planned the research. A.B.J. and S.S. performed the experiments. A.B.J. analysed the data. A.B.J. and H.G.N. wrote the manuscript.

## Supporting information


**Table S1**. Accessions devoid of latitude and longitude coordinates and thereby omitted from the WRLD dataset.
**Table S2**. ADMIXTURE and relict classifications for the WRLD_ran50 dataset.
**Table S3**. Haplotypes of additional relict accessions in the WRLD dataset.
**Figure S1.** Identification of 5′UTR *LHY* SNPs.
**Figure S2.** *LHY* 5′UTR region pY and SUA consensus sequence binding sites analysis.
**Figure S3.** Distribution of haplotypes at the country level.
**Figure S4.** Country specific population structures.
**Figure S5.** Prevalence of haplotypes in the datasets.
**Figure S6.** Haplotypes correlate with bioclimatic variables.
**Figure S7.** Cumulative variance of Principal Components.
**Figure S8.** Correlation of haplotypes with annual mean temperature.
**Figure S9.** Correlation of haplotypes with precipitation bioclimatic variables.
**Figure S10.** Correlation of haplotypes with annual precipitation).
**Figure S11.** Correlations of population distributions with low temperature and high precipitation climatic variables.
**Figure S12.** Location and features of accessions selected for isoform specific expression analysis.

This Supporting information file is the ‘WRLD’ dataset and is a CSV file containing genotype, haplotype, and bioclimatic variable data for 932 Arabidopsis accessions. The dataset is set out as 932 rows (accessions) by 48 columns (variables) of data.

This Supporting information file is the ‘WRLD_ran50’ dataset and is a CSV file containing genotype, haplotype, and bioclimatic variable data for 200 Arabidopsis accessions. The dataset is set out as 200 rows (accessions) by 48 columns (variables) of data.
