# Supplementary material for: Global spatial analysis of Arabidopsis natural variants implicates 5′UTR splicing of LATE ELONGATED HYPOCOTYL in responses to temperature
Source: Plant Cell Environ. 2018 Apr 15;41(7):1524–38. doi: 10.1111/pce.13188 (PMC6033021; doi:10.1111/pce.13188)
Supplement: Supplementary file 1 — Table S1. Accessions devoid of latitude and longitude coordinates and thereby omitted from the WRLD dataset. Table S2. ADMIXTURE and relict classifications for the WRLD_ran50 dataset. Table S3. Haplotypes of additional relict accessions in the WRLD dataset. Figure S1. Identification of 5′UTR LHY SNPs. Figure S2. LHY 5′UTR region pY and SUA consensus sequence binding sites analysis. Figure S3. Distribution of haplotypes at the country level. Figure S4. Country specific population structures. Figure S5. Prevalence of haplotypes in the datasets. Figure S6. Haplotypes correlate with bioclimatic variables. Figure S7. Cumulative variance of Principal Components. Figure S8. Correlation of haplotypes with annual mean temperature. Figure S9. Correlation of haplotypes with precipitation bioclimatic variables. Figure S10. Correlation of haplotypes with annual precipitation). Figure S11. Correlations of population distributions with low temperature and high precipitation climatic variables. Figure S12. Location and features of accessions selected for isoform specific expression analysis. [file PCE-41-1524-s001.docx]

***Plant, Cell & Environment* Supporting information**

**Article title:** Global spatial analysis of Arabidopsis natural variants implicates 5’UTR splicing of *LATE ELONGATED HYPOCOTYL* in responses to temperature

**Short running title:** Natural variation in clock temperature sensing

**Authors:** Allan B. James, Stuart Sullivan and Hugh G. Nimmo

The following Supporting information is available for this article:

**Supporting information Tables S1-S3:**

**Table S1**. Accessions devoid of latitude and longitude coordinates and thereby omitted from the WRLD dataset.

**Table S2**. ADMIXTURE and relict classifications for the WRLD_ran50 dataset.

**Table S3**. Haplotypes of additional relict accessions in the WRLD dataset.

**Supporting information Figures S1-S12:**

**Figure S1.** Identification of 5’UTR *LHY* SNPs.

**Figure S2.** *LHY* 5’UTR region pY and SUA consensus sequence binding sites analysis.

**Figure S3.** Distribution of haplotypes at the country level.

**Figure S4**. Country specific population structures.

**Figure S5.** Prevalence of haplotypes in the datasets.

**Figure S6.** Haplotypes correlate with bioclimatic variables.

**Figure S7.** Cumulative variance of Principal Components.

**Figure S8.** Correlation of haplotypes with annual mean temperature.

**Figure S9.** Correlation of haplotypes with precipitation bioclimatic variables.

**Figure S10.** Correlation of haplotypes with annual precipitation).

**Figure S11.** Correlations of population distributions with low temperature and high precipitation climatic variables.

**Figure S12.** Location and features of accessions selected for isoform specific expression analysis.

**Supporting information Datasets**: files ‘LHY SNP WRLD dataset.csv’ and ‘LHY SNP WRLD_ran50 dataset.csv’ contain the ‘WRLD’ and the ‘WRLD_ran50’ datasets, respectively. The column headers for 932 accessions (WRLD) and 200 accessions (WRLD_ran50) are:

[1] "CS_number" "tg_ecotypeid" "name.x" "country.x"

[5] "long" "lat" "collector.x" "seq_by.x"

[9] "SNP37437" "SNP37268" "SNP37245" "SNP37138"

[13] "SNP37072" "genotype" "SNP37437num" "SNP37268num"

[17] "SNP37245num" "SNP37138num" "SNP37072num" "SNPsum"

[21] "SNP37437comp" "SNP37268comp" "SNP37245comp" "SNP37138comp"

[25] "SNP37072comp" "genotype_comp" "BIO6" "BIO2"

[29] "BIO3" "BIO5" "BIO1" "BIO4"

[33] "BIO7" "BIO8" "BIO9" "BIO10"

[37] "BIO11" "BIO12" "BIO13" "BIO14"

[41] "BIO15" "BIO16" "BIO17" "BIO18"

[45] "BIO19" "altitude_GPS" "group" "relict"

**Table S1**. Accessions devoid of latitude and longitude coordinates and thereby omitted from the WRLD dataset.

|  |  |  |  |  |  |
| --- | --- | --- | --- | --- | --- |
| Ecotype ID | Name | CS No. | Country | Latitude | Longitude |
|  |  |  |  |  |  |
|  |  |  |  |  |  |
| 7236 | Litva | CS76543 | LTU | - | - |
|  |  |  |  |  |  |
| 7427 | Ko-2 | CS76531 | DEN | - | - |
|  |  |  |  |  |  |
| 7460 | Da(1)-12 | CS76470 | CZE | - | - |
|  |  |  |  |  |  |
| 7471 | RLD-1 | CS76588 | UNK | - | - |
|  |  |  |  |  |  |
|  |  |  |  |  |  |

**Table S2**. ADMIXTURE and relict classifications for the WRLD_ran50 dataset. Accession groups are colour coded according to haplotype cohort. ADMIXTURE group and relict assignations were obtained from The 1001 Genomes ADMIXTURE Map (http://1001genomes.github.io/admixture-map/#!/map). Boxed accessions are those present in the Iberian relict accessions of Table S3 of (Consortium, 2016) and includes the Can-0 line from the Canary Islands. Other Iberian ‘non-relict’ A/U/G/C/A accessions that are mapped in Figure S4d are highlighted in bold type font in the ‘country’ column.

| CS_number | tg_ecotypeid | name | country | Haplotype | ADMIXTUREgroup | relict |
| --- | --- | --- | --- | --- | --- | --- |
| CS76437 | 6680 | ANH-1 | GER | A/ G/ U/ G/ A/ | western_europe | |
| CS76457 | 7031 | Bsch-0 | GER | A/ G/ U/ G/ A/ | germany |  |
| CS76469 | 7092 | Com-1 | FRA | A/ G/ U/ G/ A/ | western_europe | |
| CS76479 | 7117 | El-0 | GER | A/ G/ U/ G/ A/ | germany |  |
| CS76490 | 6919 | Ga-0 | GER | A/ G/ U/ G/ A/ | central_europe | |
| CS76524 | 7202 | Kb-0 | GER | A/ G/ U/ G/ A/ | germany |  |
| CS76528 | 7199 | Kl-5 | GER | A/ G/ U/ G/ A/ | germany |  |
| CS76549 | 7250 | Me-0 | GER | A/ G/ U/ G/ A/ | germany |  |
| CS76611 | 7353 | Tha-1 | NED | A/ G/ U/ G/ A/ | admixed |  |
| CS76700 | 9523 | IP-Ben-0 | ESP | A/ G/ U/ G/ A/ | admixed |  |
| CS76711 | 9525 | IP-Bis-0 | ESP | A/ G/ U/ G/ A/ | spain |  |
| CS76717 | 9826 | IP-Bor-0 | ESP | A/ G/ U/ G/ A/ | western_europe | |
| CS76719 | 9827 | IP-Bos-0 | ESP | A/ G/ U/ G/ A/ | western_europe | |
| CS76742 | 9530 | IP-Car-1 | ESP | A/ G/ U/ G/ A/ | admixed |  |
| CS76754 | 9689 | Castelfed-3-205 | ITA | A/ G/ U/ G/ A/ | central_europe | |
| CS76769 | 9705 | Choto-1 | BUL | A/ G/ U/ G/ A/ | italy_balkan_caucasus | |
| CS76784 | 9838 | IP-Cot-0 | ESP | A/ G/ U/ G/ A/ | admixed |  |
| CS76864 | 6024 | Fly2-2 | SWE | A/ G/ U/ G/ A/ | south_sweden | |
| CS76900 | 9815 | Ha-HBT3-11 | GER | A/ G/ U/ G/ A/ | central_europe | |
| CS76903 | 9800 | Ha-S-B | GER | A/ G/ U/ G/ A/ | central_europe | |
| CS76904 | 9801 | Ha-SP-2 | GER | A/ G/ U/ G/ A/ | central_europe | |
| CS76925 | 9772 | Hof-1 | GER | A/ G/ U/ G/ A/ | central_europe | |
| CS76931 | 6035 | Hov1-10 | SWE | A/ G/ U/ G/ A/ | south_sweden | |
| CS76950 | 9095 | Istisu-5 | AZE | A/ G/ U/ G/ A/ | italy_balkan_caucasus | |
| CS76970 | 6040 | Kni-1 | SWE | A/ G/ U/ G/ A/ | south_sweden | |
| CS76981 | 9412 | Kor 3 | SWE | A/ G/ U/ G/ A/ | south_sweden | |
| CS76995 | 9552 | IP-Lab-7 | ESP | A/ G/ U/ G/ A/ | admixed |  |
| CS76997 | 9854 | IP-Laf-1 | ESP | A/ G/ U/ G/ A/ | western_europe | |
| CS76999 | 9102 | Lag1-4 | GEO | A/ G/ U/ G/ A/ | italy_balkan_caucasus | |
| CS77005 | 9111 | Lag2-4 | GEO | A/ G/ U/ G/ A/ | italy_balkan_caucasus | |
| CS77057 | 9782 | Lu3-30 | GER | A/ G/ U/ G/ A/ | central_europe | |
| CS77063 | 9906 | IP-Mah-6 | ESP | A/ G/ U/ G/ A/ | admixed |  |
| CS77080 | 9704 | Melni-2 | BUL | A/ G/ U/ G/ A/ | italy_balkan_caucasus | |
| CS77119 | 9089 | Nar-3 | AZE | A/ G/ U/ G/ A/ | italy_balkan_caucasus | |
| CS77121 | 9091 | Nar-5 | AZE | A/ G/ U/ G/ A/ | italy_balkan_caucasus | |
| CS77164 | 2171 | Paw-26 | USA | A/ G/ U/ G/ A/ | germany |  |
| CS77213 | 9576 | IP-Rev-0 | ESP | A/ G/ U/ G/ A/ | admixed |  |
| CS77244 | 9582 | IP-Ses-0 | ESP | A/ G/ U/ G/ A/ | spain |  |
| CS77272 | 9757 | Staro-1 | SRB | A/ G/ U/ G/ A/ | italy_balkan_caucasus | |
| CS77310 | 6125 | T710 | SWE | A/ G/ U/ G/ A/ | south_sweden | |
| CS77316 | 6132 | T790 | SWE | A/ G/ U/ G/ A/ | south_sweden | |
| CS77325 | 6148 | T960 | SWE | A/ G/ U/ G/ A/ | south_sweden | |
| CS78030 | 6077 | Rev-3 | SWE | A/ G/ U/ G/ A/ | south_sweden | |
| CS78791 | 5800 | UKID96 | UK | A/ G/ U/ G/ A/ | germany |  |
| CS78820 | 9471 | Ull-A-1 | SWE | A/ G/ U/ G/ A/ | south_sweden | |
| CS78882 | 9644 | Zupan-1 | CRO | A/ G/ U/ G/ A/ | central_europe | |
| CS78898 | 7125 | Er-0 | GER | A/ G/ U/ G/ A/ | germany |  |
| CS78914 | 9571 | IP-Pro-0 | ESP | A/ G/ U/ G/ A/ | western_europe | |
| CS78923 | 14312 | Kos-1 | RUS | A/ G/ U/ G/ A/ | asia |  |
| CS78927 | 14314 | Radk-1 | RUS | A/ G/ U/ G/ A/ | asia |  |
| CS76381 | 10010 | Sij-4 | UZB | A/ U/ G/ C/ A/ | asia |  |
| CS76411 | 9944 | Don-0 | **ESP** | A/ U/ G/ C/ A/ | relict | Y |
| CS76421 | 9957 | Borsk-2 | RUS | A/ U/ G/ C/ A/ | asia |  |
| CS76453 | 5837 | Bor-1 | CZE | A/ U/ G/ C/ A/ | central_europe | |
| CS76487 | 9762 | Etna-2 | ITA | A/ U/ G/ C/ A/ | admixed | Y |
| CS76537 | 6931 | Kz-9 | KAZ | A/ U/ G/ C/ A/ | asia |  |
| CS76560 | 772 | Neo-6 | TJK | A/ U/ G/ C/ A/ | asia |  |
| CS76594 | 7323 | Rubezhnoe-1 | UKR | A/ U/ G/ C/ A/ | asia |  |
| CS76660 | 9518 | IP-Alm-0 | **ESP** | A/ U/ G/ C/ A/ | spain | Y |
| CS76687 | 9613 | Balan-1 | RUS | A/ U/ G/ C/ A/ | italy_balkan_caucasus | |
| CS76740 | 7063 | Can-0 | **ESP** | A/ U/ G/ C/ A/ | relict | Y |
| CS76759 | 9832 | IP-Cat-0 | **ESP** | A/ U/ G/ C/ A/ | relict | Y |
| CS76763 | 9533 | IP-Cem-0 | **ESP** | A/ U/ G/ C/ A/ | relict | Y |
| CS76777 | 9836 | IP-Cod-0 | **ESP** | A/ U/ G/ C/ A/ | spain |  |
| CS76780 | 9837 | IP-Con-0 | **ESP** | A/ U/ G/ C/ A/ | relict | Y |
| CS76876 | 9125 | Geg-14 | ARM | A/ U/ G/ C/ A/ | asia |  |
| CS76886 | 9543 | IP-Gra-0 | **ESP** | A/ U/ G/ C/ A/ | relict | Y |
| CS76942 | 9851 | IP-Hue-3 | **ESP** | A/ U/ G/ C/ A/ | western_europe | |
| CS76943 | 9549 | IP-Hum-2 | **ESP** | A/ U/ G/ C/ A/ | relict | Y |
| CS76978 | 9626 | Kolyv-3 | RUS | A/ U/ G/ C/ A/ | asia |  |
| CS76980 | 9628 | Kolyv-6 | RUS | A/ U/ G/ C/ A/ | asia |  |
| CS77055 | 9554 | IP-Lso-0 | **ESP** | A/ U/ G/ C/ A/ | relict | Y |
| CS77105 | 9869 | IP-Moj-0 | **ESP** | A/ U/ G/ C/ A/ | relict | Y |
| CS77108 | 9508 | IP-Mos-1 | POR | A/ U/ G/ C/ A/ | admixed |  |
| CS77117 | 9871 | IP-Nac-0 | **ESP** | A/ U/ G/ C/ A/ | relict | Y |
| CS77129 | 9564 | IP-Nog-17 | **ESP** | A/ U/ G/ C/ A/ | spain |  |
| CS77131 | 9636 | Noveg-1 | RUS | A/ U/ G/ C/ A/ | asia |  |
| CS77152 | 9565 | IP-Orb-10 | **ESP** | A/ U/ G/ C/ A/ | admixed |  |
| CS77161 | 9607 | Panik-1 | RUS | A/ U/ G/ C/ A/ | asia |  |
| CS77165 | 9877 | IP-Pdl-0 | **ESP** | A/ U/ G/ C/ A/ | admixed |  |
| CS77169 | 9879 | IP-Per-0 | **ESP** | A/ U/ G/ C/ A/ | relict | Y |
| CS77189 | 9885 | IP-Prd-0 | **ESP** | A/ U/ G/ C/ A/ | spain |  |
| CS77196 | 9887 | IP-Pun-0 | **ESP** | A/ U/ G/ C/ A/ | relict | Y |
| CS77249 | 9745 | Sij 1/96 | UZB | A/ U/ G/ C/ A/ | asia |  |
| CS77341 | 6969 | Tamm-27 | FIN | A/ U/ G/ C/ A/ | north_sweden | |
| CS78815 | 9737 | Ulies-1 | ROU | A/ U/ G/ C/ A/ | asia |  |
| CS78840 | 9905 | IP-Ven-0 | **ESP** | A/ U/ G/ C/ A/ | relict | Y |
| CS78844 | 9598 | IP-Vim-0 | **ESP** | A/ U/ G/ C/ A/ | relict | Y |
| CS78846 | 9599 | IP-Vin-0 | **ESP** | A/ U/ G/ C/ A/ | western_europe | |
| CS78848 | 9600 | IP-Vis-0 | **ESP** | A/ U/ G/ C/ A/ | relict | Y |
| CS78864 | 9128 | Yeg-2 | ARM | A/ U/ G/ C/ A/ | asia |  |
| CS78865 | 9130 | Yeg-4 | ARM | A/ U/ G/ C/ A/ | asia |  |
| CS78866 | 9131 | Yeg-5 | ARM | A/ U/ G/ C/ A/ | asia |  |
| CS78867 | 9133 | Yeg-7 | ARM | A/ U/ G/ C/ A/ | asia |  |
| CS78868 | 9134 | Yeg-8 | ARM | A/ U/ G/ C/ A/ | asia |  |
| CS78929 | 18696 | Samm | RUS | A/ U/ G/ C/ A/ | asia |  |
| CS79018 | 7183 | Kas-1 | IND | A/ U/ G/ C/ A/ | asia |  |
| CS79036 | 19949 | OOE2-1 | AUT | A/ U/ G/ C/ A/ | central_europe | |
| CS79037 | 19950 | OOE2-2 | AUT | A/ U/ G/ C/ A/ | central_europe | |
| CS79038 | 19951 | OOE23 | AUT | A/ U/ G/ C/ A/ | central_europe | |
| CS76468 | 7077 | Co-1 | POR | G/ G/ U/ G/ A/ | italy_balkan_caucasus | |
| CS76599 | 7333 | Sei-0 | ITA | G/ G/ U/ G/ A/ | central_europe | |
| CS76634 | 768 | Zal-1 | KGZ | G/ G/ U/ G/ A/ | asia |  |
| CS76656 | 1158 | Aledal-6-49 | SWE | G/ G/ U/ G/ A/ | south_sweden | |
| CS76691 | 9619 | Basta-1 | RUS | G/ G/ U/ G/ A/ | asia |  |
| CS76706 | 9813 | BI-4 | GER | G/ G/ U/ G/ A/ | central_europe | |
| CS76713 | 9649 | Bivio-1 | ITA | G/ G/ U/ G/ A/ | italy_balkan_caucasus | |
| CS76789 | 6911 | Cvi-0 | CPV | G/ G/ U/ G/ A/ | relict | Y |
| CS76797 | 9352 | Dod 2 | SWE | G/ G/ U/ G/ A/ | south_sweden | |
| CS76810 | 5867 | Dra2-1 | SWE | G/ G/ U/ G/ A/ | south_sweden | |
| CS76863 | 6023 | Fly2-1 | SWE | G/ G/ U/ G/ A/ | south_sweden | |
| CS76867 | 9921 | FOR-23 | FRA | G/ G/ U/ G/ A/ | western_europe | |
| CS76879 | 9848 | IP-Glo-1 | ESP | G/ G/ U/ G/ A/ | spain |  |
| CS76891 | 9386 | Gron 12 | SWE | G/ G/ U/ G/ A/ | north_sweden | |
| CS76896 | 8234 | Gul1-2 | SWE | G/ G/ U/ G/ A/ | south_sweden | |
| CS76908 | 9395 | Hal-1 | SWE | G/ G/ U/ G/ A/ | south_sweden | |
| CS76955 | 9551 | IP-Jim-1 | ESP | G/ G/ U/ G/ A/ | admixed |  |
| CS76964 | 8237 | Kavlinge-1 | SWE | G/ G/ U/ G/ A/ | south_sweden | |
| CS76966 | 9770 | KBG2-13 | GER | G/ G/ U/ G/ A/ | central_europe | |
| CS77009 | 9421 | Lan 1 | SWE | G/ G/ U/ G/ A/ | south_sweden | |
| CS77010 | 9856 | IP-Lch-0 | ESP | G/ G/ U/ G/ A/ | spain |  |
| CS77015 | 9631 | Lebja-1 | RUS | G/ G/ U/ G/ A/ | asia |  |
| CS77073 | 9634 | Masl-1 | RUS | G/ G/ U/ G/ A/ | asia |  |
| CS77076 | 9866 | IP-Mdd-0 | ESP | G/ G/ U/ G/ A/ | spain |  |
| CS77089 | 9672 | Mitterberg-3-188 | ITA | G/ G/ U/ G/ A/ | central_europe | |
| CS77104 | 9868 | IP-Moe-0 | ESP | G/ G/ U/ G/ A/ | spain |  |
| CS77146 | 6071 | Omn-5 | SWE | G/ G/ U/ G/ A/ | north_sweden | |
| CS77156 | 7288 | Oy-0 | NOR | G/ G/ U/ G/ A/ | admixed |  |
| CS77167 | 9878 | IP-Pee-0 | ESP | G/ G/ U/ G/ A/ | spain |  |
| CS77211 | 6960 | Rennes-11 | FRA | G/ G/ U/ G/ A/ | western_europe | |
| CS77216 | 9577 | IP-Ria-0 | ESP | G/ G/ U/ G/ A/ | spain |  |
| CS77229 | 9578 | IP-Sac-0 | ESP | G/ G/ U/ G/ A/ | western_europe | |
| CS77258 | 9583 | IP-Sne-0 | ESP | G/ G/ U/ G/ A/ | relict | Y |
| CS77284 | 10023 | Strand-1 | NOR | G/ G/ U/ G/ A/ | admixed |  |
| CS77318 | 6134 | T810 | SWE | G/ G/ U/ G/ A/ | south_sweden | |
| CS77335 | 6172 | TAD 04 | SWE | G/ G/ U/ G/ A/ | north_sweden | |
| CS77343 | 6184 | TBO 01 | SWE | G/ G/ U/ G/ A/ | north_sweden | |
| CS77358 | 6209 | TEDEN 02 | SWE | G/ G/ U/ G/ A/ | north_sweden | |
| CS77364 | 6218 | TFA 08 | SWE | G/ G/ U/ G/ A/ | north_sweden | |
| CS77374 | 6238 | TOM 04 | SWE | G/ G/ U/ G/ A/ | north_sweden | |
| CS77375 | 6240 | TOM 06 | SWE | G/ G/ U/ G/ A/ | north_sweden | |
| CS77384 | 6244 | TRA 01 | SWE | G/ G/ U/ G/ A/ | north_sweden | |
| CS77658 | 1313 | Angso-59-422 | SWE | G/ G/ U/ G/ A/ | admixed |  |
| CS78064 | 6123 | T680 | SWE | G/ G/ U/ G/ A/ | south_sweden | |
| CS78767 | 6258 | TV-10 | SWE | G/ G/ U/ G/ A/ | admixed |  |
| CS78812 | 4900 | UKSW06-302 | UK | G/ G/ U/ G/ A/ | western_europe | |
| CS78854 | 9938 | WAV-8 | FRA | G/ G/ U/ G/ A/ | western_europe | |
| CS78858 | 7415 | Wu-0 | GER | G/ G/ U/ G/ A/ | germany |  |
| CS78917 | 6963 | Sorbo | TJK | G/ G/ U/ G/ A/ | asia |  |
| CS79000 | 4840 | UKSW06-240 | UK | G/ G/ U/ G/ A/ | western_europe | |
| CS76378 | 9956 | Stepn-1 | RUS | G/ G/ U/ G/ C/ | asia |  |
| CS76445 | 7013 | Bd-0 | GER | G/ G/ U/ G/ C/ | south_sweden | |
| CS76493 | 7147 | Gie-0 | GER | G/ G/ U/ G/ C/ | admixed |  |
| CS76535 | 7207 | Kyoto | JPN | G/ G/ U/ G/ C/ | central_europe | |
| CS76556 | 7248 | Mv-0 | USA | G/ G/ U/ G/ C/ | germany |  |
| CS76603 | 7343 | Sp-0 | GER | G/ G/ U/ G/ C/ | south_sweden | |
| CS76626 | 7394 | Wa-1 | POL | G/ G/ U/ G/ C/ | admixed |  |
| CS76633 | 7416 | Yo-0 | USA | G/ G/ U/ G/ C/ | germany |  |
| CS76645 | 9609 | Adam-1 | RUS | G/ G/ U/ G/ C/ | asia |  |
| CS76725 | 9910 | BRI-2 | FRA | G/ G/ U/ G/ C/ | western_europe | |
| CS76726 | 8231 | Bro1-6 | SWE | G/ G/ U/ G/ C/ | south_sweden | |
| CS76840 | 9843 | IP-Elp-0 | ESP | G/ G/ U/ G/ C/ | spain |  |
| CS76885 | 7158 | Gr-5 | AUT | G/ G/ U/ G/ C/ | central_europe | |
| CS76901 | 9786 | Ha-P-13 | GER | G/ G/ U/ G/ C/ | central_europe | |
| CS76944 | 9744 | Iasi-1 | ROU | G/ G/ U/ G/ C/ | admixed |  |
| CS76987 | 8240 | Kulturen-1 | SWE | G/ G/ U/ G/ C/ | south_sweden | |
| CS77052 | 7521 | Lp2-6 | CZE | G/ G/ U/ G/ C/ | central_europe | |
| CS77187 | 9707 | Podvi-1 | BUL | G/ G/ U/ G/ C/ | italy_balkan_caucasus | |
| CS77192 | 6957 | Pu2-8 | CZE | G/ G/ U/ G/ C/ | central_europe | |
| CS77194 | 9436 | Puk-1 | SWE | G/ G/ U/ G/ C/ | south_sweden | |
| CS77195 | 9437 | Puk-2 | SWE | G/ G/ U/ G/ C/ | south_sweden | |
| CS77197 | 9888 | IP-Pva-1 | ESP | G/ G/ U/ G/ C/ | spain |  |
| CS77200 | 9917 | RAD-21 | FRA | G/ G/ U/ G/ C/ | western_europe | |
| CS77209 | 9574 | IP-Rel-0 | ESP | G/ G/ U/ G/ C/ | relict |  |
| CS77255 | 2278 | SLSP-35 | USA | G/ G/ U/ G/ C/ | germany |  |
| CS77273 | 9756 | Staro-2 | SRB | G/ G/ U/ G/ C/ | central_europe | |
| CS77391 | 9794 | Tu-B1-2 | GER | G/ G/ U/ G/ C/ | central_europe | |
| CS77643 | 1070 | Brosarp-45-153 | SWE | G/ G/ U/ G/ C/ | south_sweden | |
| CS78079 | 6141 | T890 | SWE | G/ G/ U/ G/ C/ | south_sweden | |
| CS78794 | 5486 | UKNW06-233 | UK | G/ G/ U/ G/ C/ | germany |  |
| CS78805 | 5253 | UKSE06-500 | UK | G/ G/ U/ G/ C/ | western_europe | |
| CS78870 | 9747 | Zabar-1 | SRB | G/ G/ U/ G/ C/ | central_europe | |
| CS78873 | 403 | Zdarec3 | CZE | G/ G/ U/ G/ C/ | central_europe | |
| CS78879 | 9710 | Zerev-1-35 | BUL | G/ G/ U/ G/ C/ | italy_balkan_caucasus | |
| CS78890 | 9528 | IP-Cal-0 | ESP | G/ G/ U/ G/ C/ | spain |  |
| CS78913 | 6944 | NFA-8 | UK | G/ G/ U/ G/ C/ | western_europe | |
| CS78958 | 853 | MIA-1 | USA | G/ G/ U/ G/ C/ | germany |  |
| CS78960 | 867 | MIC-20 | USA | G/ G/ U/ G/ C/ | germany |  |
| CS78962 | 1612 | Brn-10 | USA | G/ G/ U/ G/ C/ | germany |  |
| CS78969 | 1741 | KBS-Mac-74 | USA | G/ G/ U/ G/ C/ | germany |  |
| CS78971 | 1757 | Ker-5 | USA | G/ G/ U/ G/ C/ | germany |  |
| CS78973 | 1797 | L-R-10 | USA | G/ G/ U/ G/ C/ | germany |  |
| CS78975 | 1820 | Lak-13 | USA | G/ G/ U/ G/ C/ | germany |  |
| CS78980 | 1942 | MNF-Che-47 | USA | G/ G/ U/ G/ C/ | germany |  |
| CS78993 | 2212 | Pent-46 | USA | G/ G/ U/ G/ C/ | germany |  |
| CS78999 | 2412 | Yng-53 | USA | G/ G/ U/ G/ C/ | germany |  |
| CS79001 | 4857 | UKSW06-257 | UK | G/ G/ U/ G/ C/ | western_europe | |
| CS79016 | 6806 | HS-17 | USA | G/ G/ U/ G/ C/ | germany |  |
| CS79025 | 7757 | KNO2.41 | USA | G/ G/ U/ G/ C/ | germany |  |
| CS79027 | 8037 | PT1.52 | USA | G/ G/ U/ G/ C/ | germany |  |

**Table S3**. Haplotypes of additional relict accessions in the WRLD dataset.

| CS_number | tg_ecotypeid | name | country | Haplotype | ADMIXTUREgroup | relict |
| --- | --- | --- | --- | --- | --- | --- |
|  |  |  |  |  |  | |
| CS76649 | 9606 | Aitba-1 | MAR | A/ G/ U/ G/ A/ | relict | |
| CS76946 | 9550 | IP-Iso-4 | ESP | G/ G/ U/ G/ A/ | relict | Y |
| CS76789 | 6911 | Cvi-0 | CPV | G/ G/ U/ G/ A/ | relict | |
|  |  |  |  |  |  | |

Figure S1


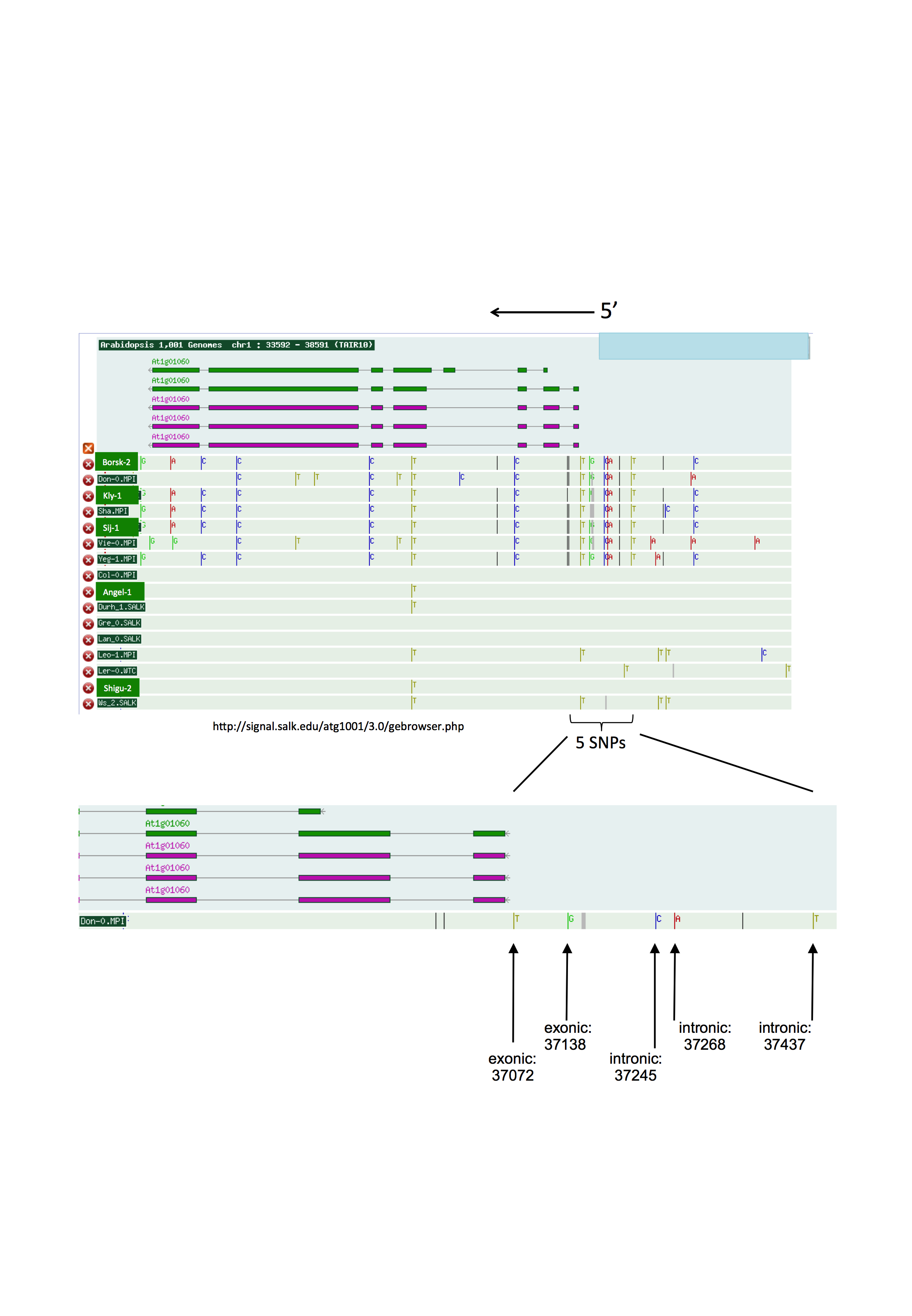


**Figure S1. Identification of 5’UTR *LHY* SNPs.** SALK 1001 genomes browser inspection of *LHY* SNPs – gene model (devoid of 5’UTR exon-intron structure) reads 3’ to 5’ (left to right) with (*upper*) a selection of accessions and their sequence SNP variations. The focus of this study has centered on the region encompassing the denoted 5 SNPs and (*lower*) their corresponding coordinates within the *LHY* gene model (the Don-0 accession was selected for the expanded region – an example of an ‘A/U/G/C/A ‘relict’ haplotype).

Figure S2

**Figure S2.** ***LHY* 5’UTR region pY and SUA consensus sequence binding sites analysis.** Polypyrimidine rich (pY) regions and SUA concensus sequence binding sites within the denoted regions of the *LHY* pre-mRNA sequence.

Figure S3

**Figure S3. Distribution of haplotypes at the country level (a)** Prevalence of haplotypes at the country level. ‘Other’ countries are detailed in panel (b). Countries denoted as their assigned ISO 3166-1 alpha-3 three-letter country codes. **(b)** Abundance and categorisation of haplotypes at the country level for countries with a low number of accessions ≤ 7 and grouped as ‘other’ countries in panel (a).

Figure S4

**Figure S4. Country specific population structures**. **(a)** Distribution of haplotypes in the U.S.A with a focus (*inset*) on haplotypes around Lake Michigan. **(b)** Distribution of haplotypes in Spain and clinal preponderance (*inset*) of haplotypes; numbers of representative accessions in each one degree latitude bin denoted adjacent to bars. **(c)** Distribution of haplotypes in Sweden and proportion (*inset*) of haplotypes within circled northern (N, accessions with latitudes ≥62, *n*=49) and southern (S, accessions with longitudes ≤14.3 and latitudes ≤56.5, *n*=113) groups. **(d)** Spanish A/U/G/C/A haplotypes are predominantly ‘relict’ variants. Spanish relict accessions in (Consortium, 2016) are projected alongside Spanish non-relicts (WRLD_50ran dataset, Table S2) and categorised for their *LHY* haplotypes. (*inset*) shows the proportion of relict lines, defined by ADMIXTURE, that are A/U/G/C/A. Map also plots Cvi-0 (Cape Verde Islands, defined as ‘relict’ by ADMIXTURE, Table S3) and Etna-2 (Italy, defined as ‘admixed’ by ADMIXTURE but also as relict in (Consortium, 2016). **(e)** Distribution of haplotypes in the UK population.

Figure S5

Figure S5. **Prevalence of haplotypes in the datasets. (a)** Prevalence of haplotypes in the WRLD and WRLD_ran50 datasets. **(b)** Latitude-longitude redundancy (instances of replicates) for the WRLD and WRLD_ran50 datasets.

Figure S6

**Figure S6. Haplotypes correlate with bioclimatic variables**. **(a)** Projection of haplotypes from the WRLD dataset onto the global index of annual mean diurnal range (BIO2, worldclim.org, index scale; °C × 10). **(b)** Principal Component Analysis (PC1 *vs* PC2) of 20 continuous variables (11 temperature (T), 8 precipitation (P) and one altitude (A) variable) and categorised for the four haplotypes for the WRLD_ran50 dataset. Box and whiskers plots (box with median and encompassing the 25^th^ to 75^th^ percentiles and extending to the min. and max. data points) of **(c)** BIO2: Annual Mean Diurnal Range (°C × 10), **(d)** BIO7: Temperature Annual Range (°C × 10), and **(e)** altitude (metres) for each haplotype cohort and 20 relict accessions defined by (Consortium, 2016) (Table S2 and S3). Pairs of means grouped by a horizontal bracket are not significantly different from each other (Tukey–Kramer method, P>0.05). Relict data are not considered for comparisons.

Figure S7


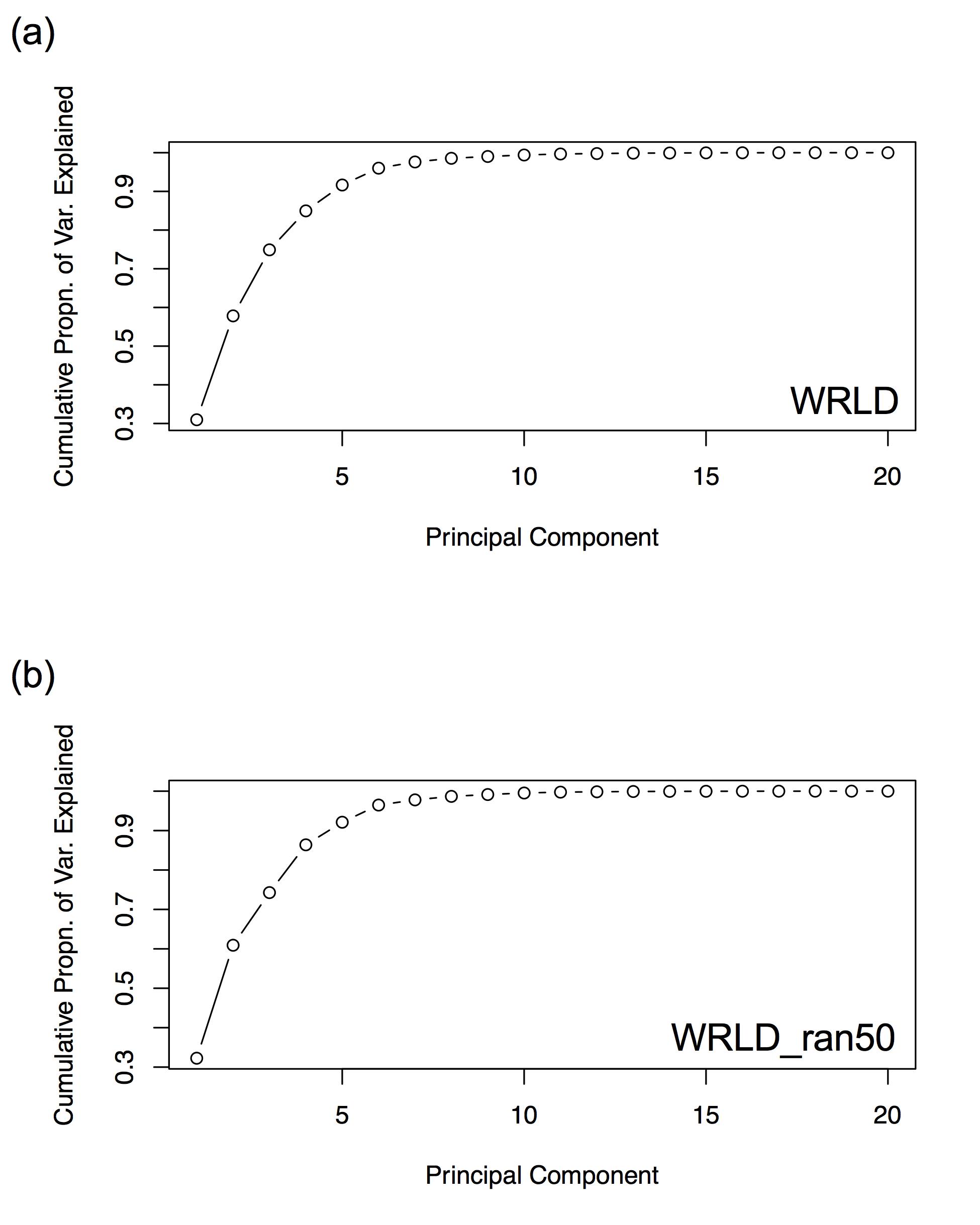


**Figure S7. Cumulative variance of Principal Components**. Plots of cumulative variance for bioclimatic and altitude principal components (PCs) for **(a)** the WRLD and **(b)** the WRLD-ran50 datasets. Six principal components explain 96 and 96.5 % of variance in the datasets, respectively.

Figure S8

**Figure S8. Correlation of haplotypes with annual mean temperature.** Means and ± SEM of BIO1: Annual Mean Temperature for each haplotype cohort for **(a)** the WRLD-ran50 and **(b)** the WRLD datasets. Pairs of means grouped by a horizontal bracket are not significantly different from each other (Tukey–Kramer method, P>0.05).

Figure S9

**Figure S9. Correlation of haplotypes with precipitation bioclimatic variables.** Means and ± SEM of **(a)** BIO14: Precipitation of the Driest Month (mm), **(b)** BIO17: Precipitation of the Driest Quarter (mm) and **(c)** BIO18: Precipitation of the Warmest Quarter (mm) for each haplotype cohort. Pairs of means grouped by a horizontal bracket are not significantly different from each other (Tukey–Kramer method, P>0.05).

Figure S10

**Figure S10. Correlation of haplotypes with annual precipitation.** Means and ± SEM of BIO12: Annual Precipitation (mm) for the WRLD_ran50 dataset. Pairs of means grouped by a horizontal bracket are not significantly different from each other (Tukey–Kramer method, P>0.05).

Figure S11


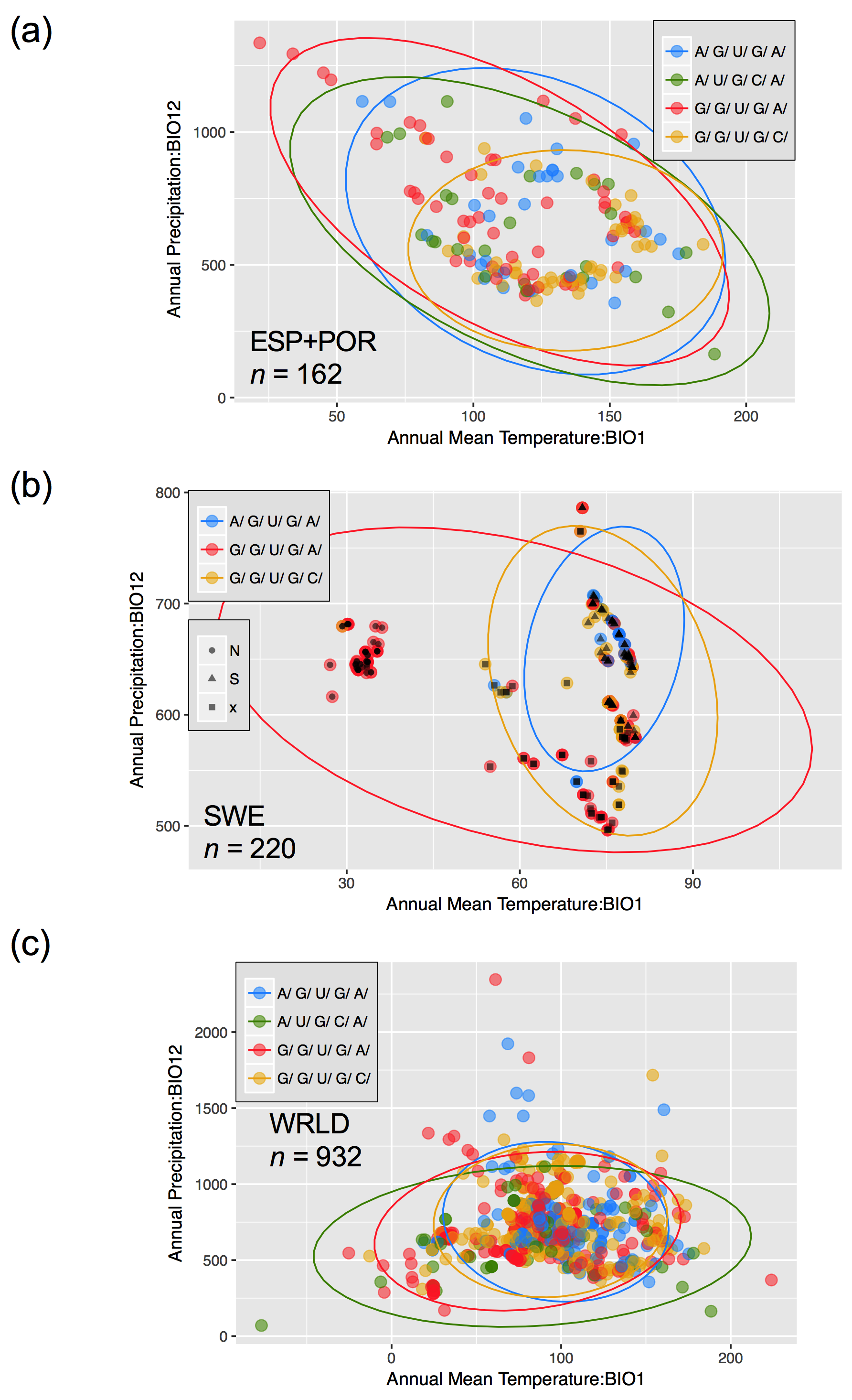


**Figure S11. Correlations of population distributions with low temperature and high precipitation climatic variables**. Scatter plots of BIO1 (Annual Mean Temperature) *vs* BIO12 (Annual Precipitation) for the **(a)** Iberian **(b)** Swedish and **(c)** WRLD datasets. Those accessions grouped as belonging to northern (N), or southern ‘Skane’ (S) populations in Figure S4(c) are denoted by shape symbol in panel (b).

Figure S12


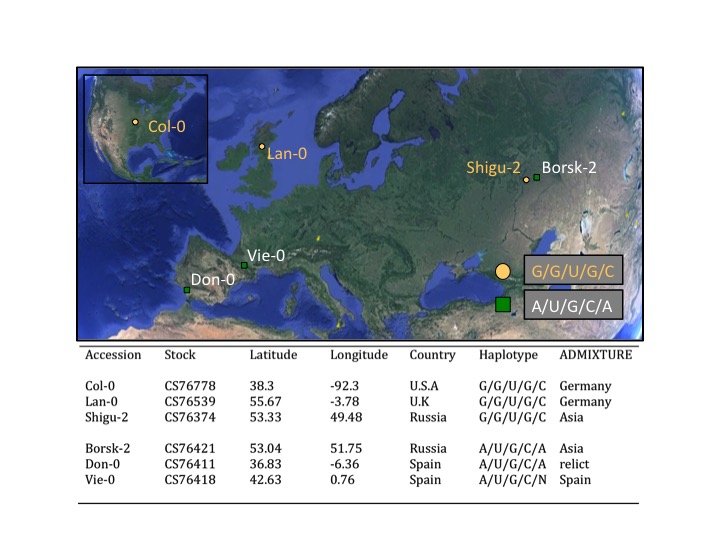


**Figure S12**. Location and features of accessions selected for isoform specific expression analysis.

Supporting Information References

Consortium T.G. (2016) 1,135 Genomes Reveal the Global Pattern of Polymorphism in Arabidopsis thaliana. In: *Cell*, pp. 481-491.
